# Study of the Electronic Structure of Coronene Doped with Nitrogen Atoms and Its Effect on CO_2_ Capture

**DOI:** 10.1021/acsomega.4c11531

**Published:** 2025-04-16

**Authors:** Kelly F. P. Laeber, Letícia M. Prates, Leonardo Baptista, Maurício T. M. Cruz

**Affiliations:** † Departamento de Química Geral e Inorgânica, Universidade do Estado do Rio de Janeiro, Rua São Francisco Xavier, 524, Maracanã, Rio de Janeiro-RJ CEP 20550-900, Brazil; ‡ Centro de Tecnologia Mineral Avenida Pedro Calmon, 900, Cidade Universitária, Rio de Janeiro-RJ CEP, 21941 908, Brazil; § Departamento de Química e Ambiental, Faculdade de Tecnologia, Universidade do Estado do Rio de Janeiro, Av. Dr. Omar Dibo Calixto Afrange, s/n—acesso pela Rod. Pres. Dutra, km 304, sentido RJ-SP—Polo Industrial, Resende, Rio de Janeiro CEP 27537 000, Brazil

## Abstract

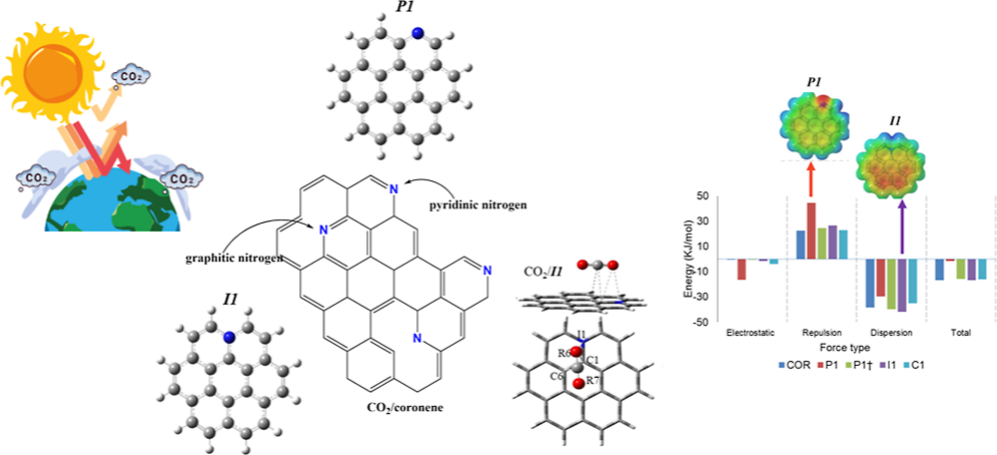

Climate change is a serious global problem. CO_2_ is of paramount importance in mitigating this environmental problem. Understanding the interaction of CO_2_ with functionalized carbon structures is essential for designing new materials to aid in efficiently capturing CO_2_. In this work, the interaction between carbon dioxide (CO_2_) and coronene models, simulating graphene and the asphaltene moiety, was studied through DFT (CAM-B3LYP-D3) and DLPNO-CCSD(T) methods to investigate the effect of nitrogen doping in two arrangements. Aromaticity, electronic, and topological properties were evaluated using HOMA, HOMO–LUMO gap, QTAIM, and NCI methods. The results show that the adsorption of CO_2_ in the coronene molecule is dependent on the position of the heteroatom and governed by noncovalent interactions, such as van der Waals and hydrogen bonds. The CO_2_/*N*-coronene complex with pyridinic-N is stabilized due to two unconventional hydrogen bonds parallel to the aromatic π system. We hope that the present results can help the synthesis of inhibitors of asphaltene precipitation and better systems for CO_2_ capture.

## 1 Introduction

Planet Earth’s temperature is predicted to increase by up to 1.5 °C by 2030, according to the International Panel on Climate Change (IPCC), if human greenhouse gas emissions continue at current levels.^1^ In recent assessment reports, the National Oceanic and Atmospheric Administration Global Monitoring Laboratory (NOAA GML) showed that the average concentration of CO_2_ at the global surface in 2023 reached a staggering value of 419.3 ppm (an increase of 2.8 pm during the year).^2^ This alarming record underscores and dramatizes the search for reducing CO_2_ emissions and their atmospheric concentration, which in its studies have been growing vertically.

While widely used as practical materials, aqueous amine solutions suffer from high parasitic energy consumption and adverse environmental impacts, solvent losses, and corrosion issues.^3^ Several materials have been developed as solid adsorbents to CO_2_, such as carbonaceous porous materials, porous organic materials, covalent organic frameworks, metal–organic frameworks, and molecular sieves.^4^ Among them, graphene, a two-dimensional all-sp^2^ carbon allotrope with remarkable properties (mechanical strength, high surface area, electrical conductivity, low density, high chemical stability, excellent processability, and eco-friendly behavior), has presented a considerably large number of studies in the past few years.^5−18^

In parallel, CO_2_ injection strategies for enhanced oil recovery seem to be a strategy for CO_2_ mitigation and better use of oil reservoirs.^19,20^ However, CO_2_ injection can induce asphaltene precipitation, clog the oil reservoir, and impact the extraction and oil field lifetime.^21−27^ Nitrogen doping has effectively tailored the graphene properties and rendered its potential use for various applications.^28−38^ An update in the graphene utilization as carbon dioxide removal technology is the introduction of nitrogen atoms producing nitrogen-doped graphene and its derivatives, such as nitrogen-doped graphene oxide. Numerous studies have documented the effectiveness of these systems in CO_2_ capture applications.^39−46^ Recent experiments have revealed a notable improvement in the CO_2_ adsorption capacity of graphenes following nitrogen doping in the pyridinic and pyrrolic forms.^30^

Theoretical studies have confirmed that incorporating nitrogen atoms into the graphene structure promotes the process of CO_2_ physisorption.^47^ Theoretical studies employing different N-graphene models have shown that the binding energy of CO_2_ is mainly driven by van der Waals force and varies in narrow ranges, as in the C_23_H_12_N···CO_2_ complex (from −3.24 kcal/mol to −3.56 kcal/mol), however, without involving significant intermolecular charge transferences.^48^ Molecular electrostatic potential (MEP) analysis shows that such replacements increase the molecules’ electron-rich environments.^40^

In the asphaltene case, the correct structure is unknown, and only knowledge of the element’s proportions, the average structure, and the presence of free radicals is available.^49,50^ Nitrogen takes part in the aromatic portion of asphaltenes, but this role in CO_2_ interaction and precipitation is barely described.^51−53^ The study of the influence of heteroatoms on aggregation is of utmost importance since their role in aggregation is still unclear. Some studies available in the literature^54−57^ indicate that an increase in their content can promote an imbalance in the charge density inducing permanent or partial dipoles absent in nonpolar species^49,55^ and thus contribute to the increase in the aggregation phenomenon forming stable complexes. Although the number of aromatic rings rules the self-aggregation process, heteroatoms in the aromatic moiety and the aliphatic side chains are another determining factor.^54,58^ Furthermore, depending on the position in the structure, they can induce different interactions with low-polarity organic molecules and favor intermolecular interactions.^59^ Knowledge of these characteristics eases aggregation control, optimizing work on an oil production line. Also, the discussed properties may help the synthesis of inhibitors of aggregation, which may be alkaline or acidic, according to the asphaltene characteristics..^52^

The effect of graphitic-N (or Y-carbon due to structural arrangement) and pyridinic-N in the reactivity of graphene and asphaltene was studied by doping the coronene molecule. The doping effect on CO_2_ adsorption was done by approaching a single CO_2_ molecule to substituted and nonsubstituted coronene. Quantum chemical methods such as DFT and DLPNO-CCSD(T) were employed in the entire study. The literature shows a previous study concerning the interaction of CO_2_ with the pyridine arrangement,^31^ but there are no reports with the other substitutions considered here, especially about asphaltenes and CO_2_.

Coronene has recently attracted great attention from the perspective of materials science because of its relation to graphene and being a good asphaltene prototype.^31,60−63^ Coronene is a π-planar polycyclic aromatic hydrocarbon (PAH) molecule with *D*_6h_ symmetry. It is the smallest homologue of benzene with six-fold symmetry and the smallest fragment of graphene, in which all C–C bonds of the central ring are shared with the peripheral rings. For these reasons, coronene is called superbenzene or nanographene.^64^

The work presented herein concerns properties evaluating substituted and nonsubstituted coronene and its complex with CO_2_. The N+doping effect in coronene’s frontier orbitals and reactivity parameters are evaluated. DFT-D3 and DLPNO-CCSD(T) methods are employed to assess the geometric and energetic parameters. The electronic structure, such as charge distribution, aromaticity, and topological properties of the considered systems, will be discussed to assess how planar aromatic structure interacts with CO_2_, giving insights about CO_2_ capture and asphaltene aggregation.

## 2 Methodology

The methodology was chosen by their good results in describing high molecular weight aromatic structures, such as graphene sheets.^65,66^ The CAM-B3LYP methodology, with the addition of the D3 dispersion correction and the 6-311G(d,p) basis set, presented a result for the interaction energy between a pair of coronenes in good agreement with Coupled Cluster (CCSD) calculations (16.19 kcal mol^–1^ versus 17.67 kcal mol^–1^).^67^ Thus, this level of theory was used to optimize all coronene structures considered in this work. In addition, the DLPNO-CCSD(T) method was used to refine the electronic energy and test the wave function multiconfigurational character via T1 diagnosis in each isolated structure.^68^ In the case of complexes, the DLPNO-CCSD(T) calculation was conducted to check the precision of DFT results. The molecular structure of coronene was constructed from the Gaussview 6.0 package.^69^

All geometry optimizations were performed at the DFT level, without imposing symmetry, employing a hybrid exchange-correlation functional using the Coulomb attenuating method^70^ and Grimme’s dispersion correction scheme,^71^ CAM-B3LYP-GD3, and 6-311G(d,p) basis set. The wave function stability was tested to check if the system was in the right spin state. The minimum energy structures were confirmed by using vibrational frequency calculations. These steps were performed using the Gaussian 16 software.^72^

The interaction energy (*E*_int_) for each CO_2_/coronene complex was calculated (eq 1) and posteriorly corrected using the counterpoise method^73^ to eliminate the basis set superposition error (BSSE).
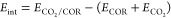
1*E*_CO_2_/COR_, *E*_COR_, and E_CO_2__ represent the absolute energies of the CO_2_/coronene (or CO_2_/N-coronene) complex, coronene, and CO_2_ molecule, respectively.

**Figure 1:**
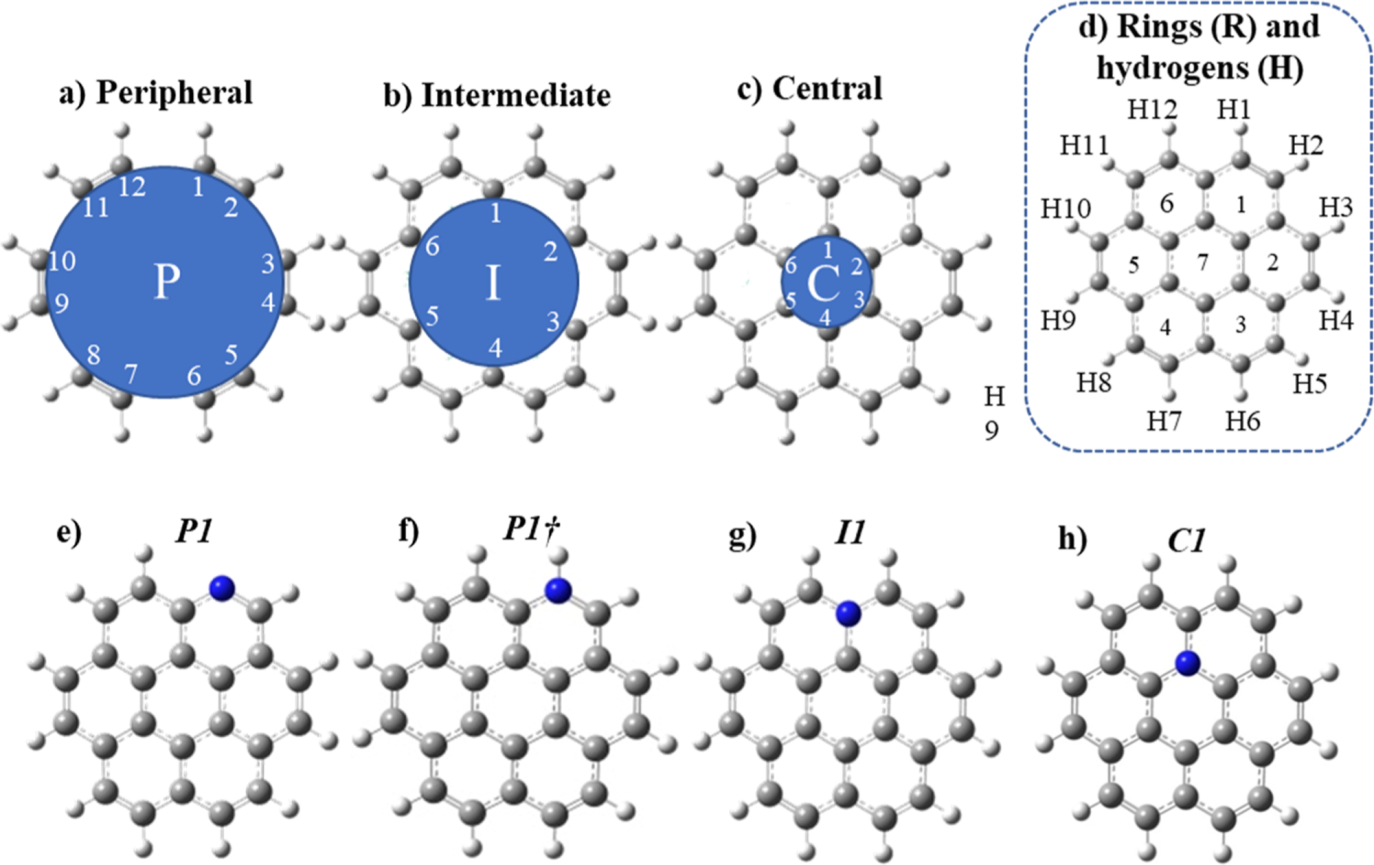
Atomic labels scheme with clockwise numbering in the (a) peripheral (P), (b) intermediate (I), and (c) central (C) regions as well as (d) rings (R) and hydrogen atoms (H). Also, N-coronene structures (e–h) and their respective notations (***P1***, ***P1†***, ***I1***, and ***C1***).

All single-point DLPNO-CCSD(T) calculations were carried out using cc-pVDZ basis sets, cc-pVDZ auxiliary basis set, and resolution of the identity approach using the ORCA 4.2.1 suite of programs.^74^ All considered molecules showed no multiconfigurational character, so DFT methods can be applied well to study the proposed systems. Further, a set of structural (bond length and bond and dihedral angles), electronic (HOMO–LUMO gap, charge distribution, electrostatic potential maps, and QTAIM analysis), and energetic parameters were used to evaluate the obtained structures. The atomic charges were calculated using Chelpg,^75^ Merz–Signh–Kollman,^76^ Hirshfeld,^77^ and NBO^45^ models. A QTAIM analysis for each structure was performed using the AIMAll software version 19.10.12.^78^ QTAIM was used to calculate the critical bond points (BCP), critical ring points (RCP), and the Laplacians of the density (∇^2^) of the structures and their interaction with the CO_2_ molecule. Considering the complexes’ optimized geometries, the Wiberg binding indices (WBIs) were calculated in the NAO basis as contributions from atomic orbital pairs using Multiwfn 3.8.^79,80^ The aromaticity of the models was evaluated by using two criteria: (i) geometric: represented by the Harmonic Oscillator Model of Aromaticity (HOMA) method and (ii) energetic: represented by the HOMO–LUMO gap. HOMA^81^ was calculated using Multiwfn 3.8^79^ according to the numbering shown in Figure 1a (green numbers) and eq 2.
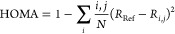
2where *N* is the number of the atoms considered, *j* denotes the atom next to atom *I*, and α and *R*_ref_ are precalculated constants given in the original paper^82^ for each type of atom pair.

The interactions between the coronene structures and the CO_2_ molecule were assessed by the noncovalent interactions approach (NCI),^79,83,84^ and the energy decomposition method based on the molecular force field (EDA-FF) was also used.^79,85,86^ The reduced density gradient and the sign  are functions that together are fundamental to identifying these regions and are connected with eq 3
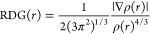
3where ρ(*r*)e and ∇ρ(*r*) are the density and electron density gradient at position *r*, respectively. In the *s* versus ρ graphs, the NCI is associated with the characteristic peaks appearing in regions of low electron density caused by decreasing the density gradient at these points. The EDA-FF was performed through the AMBER force field using the Multiwfn software 3.8.^79^

Also, Born–Oppenheimer molecular dynamics (BOMD) were conducted for the CO_2_–coronene complexes at the same level as the optimization process theory. The initial temperature was chosen as 100 °C because it is the average temperature of reservoir oils, and coronene is considered a good asphaltene model. After full Hessian calculation, the initial thermal energy was randomized between all vibrational and rotational levels. The simulation was performed up to 500 fs, updating the Hessian every 10 simulation steps. One trajectory was performed for three different structures. All calculations were performed at the Gaussian 16 package.^72^

## 3 Results and Discussion

### 3.1 Structural Analysis: Coronene and N-Coronene

The coronene structure has been used as a model for the graphene structure and its derivatives in theoretical calculations.^31,60−63^ This model helps understand the gas interaction mechanism with a local environment presenting an aromatic structure.^87^ Although coronene is a good simplified model of graphene fragments, it may not fully replicate its extended electronic structure and adsorption properties. Kim and collaborators^88^ employed DFT methods with dispersion corrections to analyze the binding energies and bond distances between halogen molecules (Cl_2_ and Br_2_) with aromatic systems such as benzene and graphene. Also, one can evaluate how the interaction varies with the size of the aromatic system. They found that as the size of the aromatic system increases, electrostatic interactions become less significant, while dispersion interactions increase. However, coronene still presents significant advantages as a model for graphene, especially due to its lower computational cost. It is finite and symmetric, allowing faster and more efficient calculations with DFT methods, providing suitable approximations for graphene’s electronic properties. Coronene is a planar PAH that resembles a graphene moiety. It maintains the extended π system, which is paramount for gas adsorption. Coronene can be synthesized and functionalized more easily than graphene, allowing more controllable experimental tests.^31^ It is also used as a model for asphaltenes and other carbon systems, facilitating studies on CO_2_ adsorption and pollutant capture.^89−91^ This allows coronene-based studies to provide insights into interactions of molecules with carbon surfaces.^92−94^

The nitrogen substitution in PAH structures, such as coronene, can significantly impact their electronic properties, mainly because the nitrogen atom contains one more valence electron than the carbon atom. The substitution changes HOMO–LUMO gaps, absorption and emission properties, redox potentials, basicity, and more. Therefore, investigating the influence of the position and arrangement of nitrogen atoms within PAHs is a motivating and necessary task.^95−97^ In 6-fold rings, nitrogen doping can occur in two sp^2^ arrangements: pyridinic and graphitic. In the pyridinic arrangement, the nitrogen atom is placed at the edge of the structure or in a defect. Pyridinic-N uses two valence electrons to form two σ-bonds with two adjacent carbon atoms and adds a third electron to the aromatic π system.^98^ The remaining two electrons stay in the nitrogen atom as lone pair electrons in the orbital oriented along the graphene plane. In the graphitic arrangement, the nitrogen atom replaces a carbon atom within the internal structure, yielding three σ-bonds with three neighboring carbon atoms. These arrangements maintain the highest structural similarity between N-coronene and coronene and change the electronic configuration of the N-doped system, leading to a doublet multiplicity as the electronic ground state. Because of this, this work did not include other arrangements, such as pyrrolic, aminic, or nitrilic.

Therefore, a single nitrogen atom was then incorporated, replacing a carbon atom in coronene, generating four distinct configurations: pyridinic (***P1***),^31^ pyridinium ion (***P1†***), and two graphitic (***I1*** and ***C1***) (Figure 1d–g, respectively). The coronene structure was divided into three different regions to ease the recognition of each atom: peripheral (P), intermediary (I), and central (C) or as known in the literature Y-carbons.^99,100^ They were numbered following the clockwise direction: from 1 to 12, from 1 to 6, and from 1 to 6, respectively (Figure 1a–c). The hydrogen atoms follow the same numbering given to the peripheral atoms (Figure 1a), and the rings were also numbered from 1 to 7 (Figure 1h). This systematic and explicit notation, which includes the initial letter of the region’s name and the respective number, ensures each atom’s easy and precise identification. For instance, P12 is the atom of label 12, located in the peripheral region (P). Therefore, the range of atoms by region follows P1–P12 (peripheral), I1–I6 (intermediary), and C1–C6 (central), whereas H1–H12 and R1–R7 refer to the sequence of hydrogen atoms and aromatic rings, respectively, as depicted in Figure 1a–c below.

The notation given to the coronene structure is ***COR***. In contrast, to the N-coronene structures, it is simply the identification of the nitrogen atom position but in *italics* and **bold** format: ***P1***, ***P1†***, ***I1***, and ***C1*** (where the ***†*** symbol indicates pyridinium-N), as shown in Figure 1e–h.

The search of the preferential electronic spin state indicates both ***COR*** and ***P1*** (N-pyridinic) are singlets, whereas ***I1*** and ***C1*** (both graphitic-N (or Y-carbon)), and ***P1†*** (N-pyridinium) are duplets. At first sight, ***I1*** and ***C1*** can be considered to be cationic or triplet structures. However, the wave function stability analysis showed that these structures have a duplet ground state. Preliminary tests showed that nitrogen insertion into I1 and C1 positions leads to open-shell structures with lower energies than charged ones. A second reason to investigate the neutral radicals is that asphaltenes present free-radical characteristics in their composition and noncharged species.^101,102^ The study about this radicalar character can help understand asphaltene aggregation, a well-known issue of the oil extraction process.^26,102,103^

The optimized structure of ***COR*** presents C–C bonds whose lengths depend on the region. According to Table S1 and Graphs S1–S3 (in Supporting Information), the C–C bond length is greater in the central than the peripheral region in the coronene molecule with *D*_6h_ symmetry (1.423 Å against 1.361 Å). The C–C bonds connecting the ring regions present intermediary values and increase in opposite directions from the central ring to the peripheral region: central-intermediary (1.408 Å) < intermediary-peripheral (1.420 Å). The C–H bond lengths present a value of 1.084 Å. The C–C–C bond angle values vary depending on the region where the carbon atoms are located. Evaluating by rings, in R1, the six C–C–C bond angles values are P1–P2–I2 and I1–P1–P2 (symmetric) = 121.169°, P2–I2–C2 and C1–I1–P1 (symmetric) = 118.831°, and I2–C2–C1 and C2–C1–I1 (symmetric) = 120.000°. Because of *D*_6h_ symmetry, the same values are seen in the R2, R3, R4, R5, and R6 rings. In the central ring, R7, all values are 120.000°. Two different C–C–H bond angle values are present: P1–P2–H2 = 120.215° and I2–P2–H2 = 118.616°. All dihedral angles are 0° or 180°, indicating that the structure of the coronene molecule remains perfectly planar after optimization of its coordinates. Such obtained geometric parameters present very similar values to those obtained with different functionals added with Grimme’s dispersion correction, using the same 6-311G(d,p) basis (herein employed) for coronene, such as wB97X-D.^104^

The atomic coordinates of ***COR*** undergo minimal changes after substituting a carbon with a nitrogen atom, as detailed in Table S1 (Supporting Information). The C–N bond lengths vary with the arrangement and position of the nitrogen atom. For instance, the pyridinic-N in ***P1*** has the shortest C–N bond length (P1–P2) with a value of 1.301 Å, while the other bond (P1–I1) is barely elongated, with a value of 1.366 Å.

When pyridinic-N is protonated to pyridinium-N (***P1†*** ionic structure), the C–N bonds, P1–P2 and P1–I1, elongate to 1.332 Å and 1.375 Å, respectively. The graphitic-N located in the intermediary region (***I1*** structure) has two C–N bonds with length values of 1.388 Å (I1–P1 and I1–P12) and one of 1.408 Å (I1–C1). On the other hand, when the graphitic-N is in the central region (***C1*** structure), their C–N bonds become even more elongated, with two in the central ring, with values of 1.405 Å (C1–C2 and C1–C6) and another of 1.424 Å (C1–I1). Therefore, the N–C bond lengths follow the same behavior seen in the C–C bonds: they are longer in the central than the peripheral region.

A more comprehensive and detailed analysis of the changes in C–C bond lengths after nitrogen doping can be conducted by referring to Graphs S1a–c and S2a–c in the Supporting Information section. Analysis of Graph S1a reveals that the replacement of the carbon by a pyridinic nitrogen atom (***P1*** structure) results, in general, in little changes in the C–C bond lengths. The most significant changes are located in the R1 ring, where the nitrogen atom is added, and in the central ring, R7. In both cases, the C–C bonds are smaller when compared with C–C bonds in coronene, with shortening varying in the ranges of 0.005 to 0.009 and 0.04 to 0.11 Å, respectively.

The C–C bond length changes are more noticeable when the nitrogen atom is substitutional (or graphitic) than those observed in pyridinic-N or pyridinium-N. Based on Graph S2a, the substitution with graphitic-N causes more noticeable changes in almost all regions. In the I1 structure, the most significant C–C bond length changes are mainly observed in the peripheral and peripheral-intermediary regions. Specifically, there is a shortening in the peripheral region in P1–P2 and P11–P12 (symmetric), from 1.361 to 1.339 Å. Also, an elongation in P3–I2 and P9–P10 (symmetric) occurred from 1.361 to 1.380 Å. In the peripheral-intermediary region, an elongation in P2–I2 and P11–I6 (symmetric), from 1.420 to 1.445 Å, and a shortening in P3–I2 and P10–I6 (symmetric), from 1.420 to 1.394 Å, are observed. Additionally, a very slight elongation in the peripheral-central connection region is verified, in the range of 0.006–0.008 Å, along with a shortening in the central ring R7, particularly in C1–C2 and C6–C1 (symmetric), of 0.016 Å (from 1.423 to 1.407 Å).

The structure optimization procedure of the isolated N-coronene units in this work converges to fully planar geometries, as observed in the literature.^101,105,106^ All C–N bond distances obtained in this work agree with values reported in the literature for pyridinic and graphitic nitrogen atoms in naphthalene aza-derivatives.^106^

### 3.2 Frontier Molecular Orbital (FMO) Analysis

The HOMO–LUMO gap energy is an index of great relevance in predicting the stability of molecules, and the resonance energy can be measured by it.^107^ The greater difference between the HOMO–LUMO orbitals is associated with higher stability.^108−110^ Thus, this parameter should provide a dimension of stabilization promoted by aromaticity. The calculated coronene’s HOMO – LUMO gap is 6.26 eV, a value close to that found in the work of Ejuh and collaborators (7.31 eV), who adopted the TD-wB97XD/cc-pVDZ methodology.^111^ However, the literature shows that the value of the energy gap is very dependent on the methodology applied, with values in the range of 2.86 to 7.31 eV being obtained depending upon the methodology.^111−115^ The HOMO–LUMO gap of coronene with chlorine atoms also presented results sensitive to the methodology used.^115^ For these reasons, the HOMO–LUMO gap of the molecule-substituted molecules will be evaluated in comparison to coronene at the same level of theory applied here. Despite the range of values in the literature, as a role of thumb, a high gap value is associated with a high structure’s aromaticity because a greater HOMO–LUMO gap lowers the structure’s reactivity.^111^

**Figure 2:**
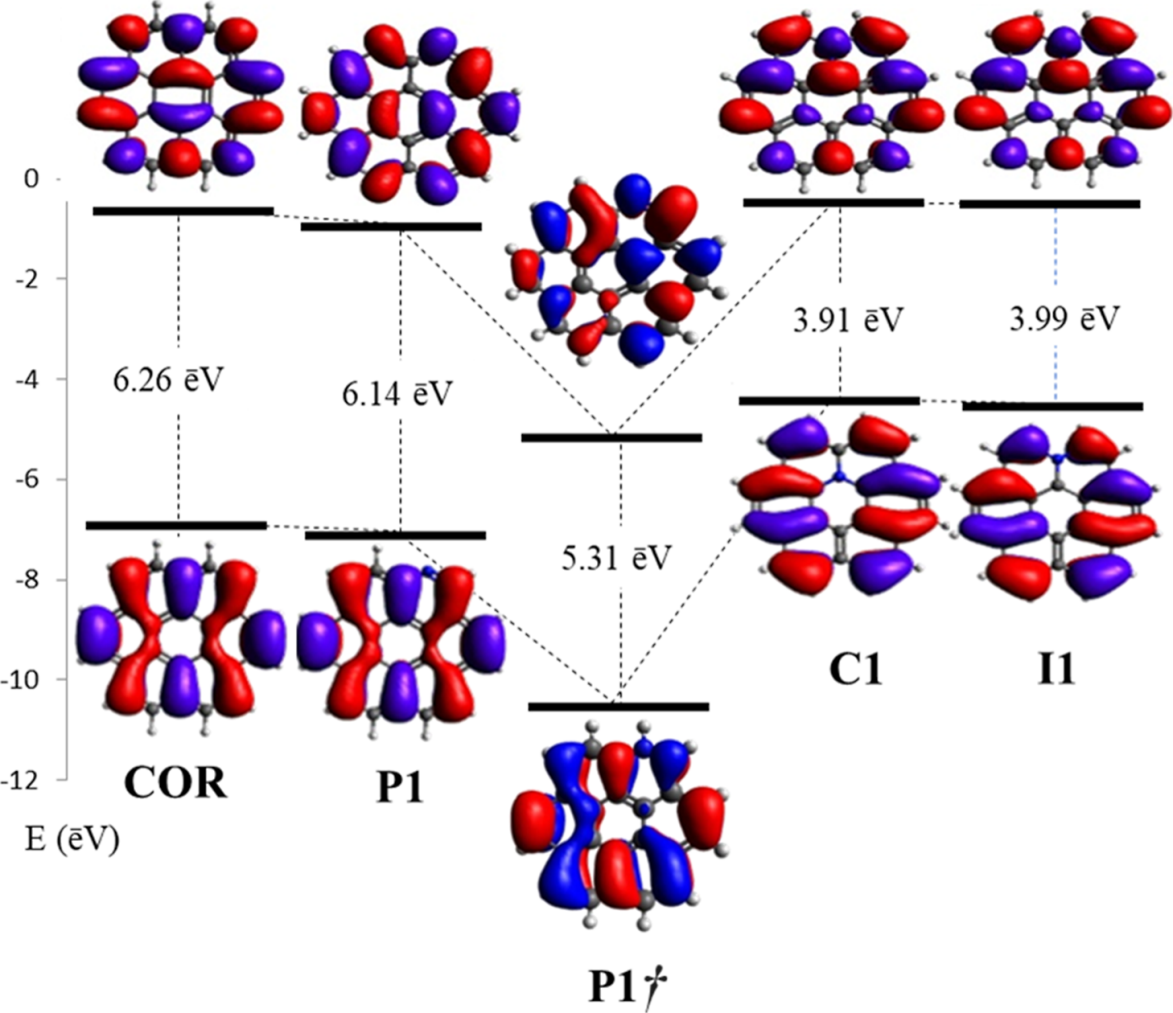
LUMO and HOMO energies and the HOMO–LUMO gap of the coronene (**COR**) and N-coronene (***P1***, ***P1†***, ***I1***, and ***C1***) structures optimized using the CAM-B3LYP/6-311G(d,p) methodology.

The frontier molecular orbital (FMO) analysis shows that pyridinic-N doping (***P1*** structure) promotes a slight lowering of energy of both LUMO and HOMO in comparison with the coronene (Figure 2). In addition, there is a slight downshifting of −0.23 eV from the midpoint of energy between the HOMO and LUMO energy [*E*_HOMO_ + 1/2(*E*_gap_)], from −3.73 eV to −3.96 eV. According to the literature, heteroatom carbon replacement results in a red shift, as observed in the present results.^116^ However, studies involving asphaltene models show a negligible effect of dopants on FMOs.^100^ In contrast, other authors used the HOMO–LUMO gap as a parameter to characterize different asphaltene molecules.^117^

The nitrogen substitution in ***I1*** and ***C1*** positions promotes an increase in the HOMO–LUMO energies, being well more pronounced on HOMO (***I1***: from −6.86 eV to −4.39 eV and ***C1***: from −6.86 eV to −4.48 eV) than LUMO (***I1***: from −0.60 eV to −0.48 eV and ***C1***: from −0.60 eV to −0.49 eV), consequently resulting in an increase in the value of the midpoint of energy between HOMO and LUMO in ***I1*** as well as ***C1***: (+1.30 eV and +1.25 eV, respectively).

These results reflect the behavior presented by an extended pristinic graphene sheet, which has a reduced work function after it is doped with graphitic-N.^1^ DFT calculations performed in an extended graphene sheet containing 1% dopants/defects indicate an average addition of 0.54 electron charge to the π network of the pristine graphene lattice by each graphitic–N bond, promoting a reduction on the work function of the graphene.

In summary, the N-substitution decreases the HOMO–LUMO gap, mainly for ***I1***, ***C1***, and ***P1†*** structures (3.91, 3.99, and 4.17 eV, respectively) compared to COR, which is indicative of lower aromaticity (higher reactivity) of these systems in comparison with COR. The subtle decrease in the ***P1*** gap (6.01 eV) suggests that the N substitution at the peripheral region has a small effect on the overall degree of aromaticity of coronene. These values are corroborated by the findings of the literature that show that aromaticity will decrease in the presence of heavier heteroatoms.^118^

### 3.3 CDFT-CDFT-Based Reactivity Descriptors Analysis

In this study, we examine how nitrogen doping affects the reactivity of coronene by analyzing various global descriptors within the conceptual density functional theory (CDFT) framework. CDFT has provided insights into various physicochemical problems over the past few decades.^119,120^ A brief overview of CDFT’s basic principles can be found in the work of Chakraborty and Chattaraj.^121^ Among the reactivity descriptors, we focused on ionization energy (IE), electron affinity (EA), global hardness (η), chemical softness (*S*), chemical potential (μ), and electrophilicity (ω). The equation used to calculate each descriptor and the obtained results for both coronene and N-coronene molecules are presented in Table 1.

**Table 1:** Global Activity Parameters for Coronene (***COR***) and N-Coronene (***P1***, ***P1†***, ***I1***, and ***C1***): Ionization Energy (IE), Electron Affinity (EA), and Global Hardness (η), All in e̅V, Chemical Softness (*S*), in e̅V^–1^, Chemical Potential (μ), in e̅V, and Electrophilicity Index (ω), Dimensionless

parameter	ionization energy (IE)	electron affinity (EA)	global hardness (η)	chemical softness (*S*)	chemical potential (μ)	electrophilicity index (ω)
equation	–*E*_HOMO_	–*E*_LUMO_	*E*_gap_/2	1/(2η)	–(IE + EA)/2	μ^2^/2η
***COR***	6.86	0.60	3.13	0.16	3.73	2.22
***P1***	7.03	0.89	3.07	0.16	3.96	2.55
***P1†***	10.43	5.12	2.65	0.19	7.78	11.38
***I1***	4.39	0.48	1.96	0.26	2.44	1.52
***C1***	4.48	0.49	2.00	0.25	2.49	1.55

A molecule’s reactivity increases as the chemical potential (μ) decreases. Chemical hardness is a parameter that characterizes a molecule’s chemical stability. A system with a high chemical hardness is considered very stable. Thus, molecules with a low chemical hardness are more reactive, polarizable, and less stable. Hardness is also a way to measure the stability of a chemical system in terms of its deformation. The electrophilicity index defines the tendency of an electrophile to acquire a given amount of electron density and the resistance for a molecule to exchange electron density with the surroundings.^122^

The ionization potential calculated for coronene is 6.86 e̅V, close to the value determined experimentally (7.29 ± 0.03 e̅V).^123^ Because of the high resemblance in the energy levels of the frontier orbitals (HOMO and LUMO) obtained for COR and P1, these two species exhibit high similarity in all of the reactivity descriptors used in the study. Therefore, it is inferred that ***P1*** has stability comparable to that of ***COR***, making it less likely to transfer charge or participate in reactions compared to other N-coronenes tested. An analogous result is also observed in smaller similar systems, such as naphthalene and azanaphthalene.^108^

The structures of ***I1*** and ***C1*** have the lowest hardness and the highest chemical potential, indicating their greatest tendency to charge transfer. The electrophilicity values follow the order: ***P1†*** > ***P1*** > ***COR*** > ***C1*** > ***I1*** (11.38, 2.55, 2.22, 1.55, and 1.52, respectively). This result suggests that, among the neutral structures, those with pyridinic-N are most capable of attracting electron density, regardless of the position occupied by the quaternary nitrogen atom. The protonated structure (pyridinium-N, ***P1†*** structure) appears in the first place because it is the species positively charged 1+. This positive charge can also explain the higher ionization potential and electron affinity of the protonated ***P1†*** structure. In evaluating global hardness, Table 1 shows the following decreasing order: ***COR*** > ***P1*** > ***P1†*** > ***C1***> ***I1***, with values of 3.13, 3.07, 2.65, 2.00, and 1.96, respectively. This result indicates that ***COR*** has the greatest chemical hardness while ***I1*** has the smallest. The order implies that coronene is the most stable, whereas coronene doped with graphitic N, located closest to the edge of the aromatic structure, is the least stable.

### 3.4 ESP Charge Distribution

The charge distribution analysis on coronene was performed using different methods: Hirschfeld, NBO, MKS, and ChelpG. According to the results, methods based on the adjustment of the electrostatic potential, such as ChelpG and Merz–Singh–Kollman (MSK), are more consistent with what should be expected for PAH structures.^124^ Among the two methods, MSK was chosen because it agrees with the literature on charge distribution results.^124^ The method calculates the partial atomic charges reproducing the molecular electrostatic potential through a least-squares fit using a grid of points around the molecule. Partial charges obtained by the electrostatic potential fit are suitable for estimating interactions between molecules, mainly of an electrostatic nature.^76^

The charge distribution analysis (see Table S2a–c and Graph S1 in the Supporting Information) on the carbon atoms in ***COR*** appoints negative charges in the peripheral carbons (from −0.187 e̅ to −0.226 e̅) and positive charges in the intermediary ones (from +0.088 e̅ to +0.135 e̅), and in the central ring, they are roughly neutral (−0.003 e̅ to +0.014 e̅). At the same time, the hydrogen atoms are in the positive charge portion (from +0.147 e̅ to +0.155 e̅). Doping with a nitrogen atom promotes modifications in the charge distribution on the coronene structure, depending on the region occupied by this atom, as observed previously in the literature for similar systems.^105^ The most significant alterations are observed when the nitrogen atom is placed in the peripheral region with a pyridinic arrangement (***P1*** structure). In this arrangement, it attracts a significant portion of the electronic density (symmetrically spread in ***COR***), carrying a negative charge of −0.700 e̅ and inducing a positive charge on the two directly bonded carbon atoms (*q*(P2) = +0.355 e̅ and *q*(I1) = +0.723 e̅). The carbon atom I1 is more electron-deficient since the partial charge changes from +0.127 e̅ (in COR) to +0.723 e̅ after substituting with N-pyridinic. In contrast, carbon (I2) becomes negatively charged (−0.272e̅). The central ring is the most affected by the substitution because the charges change considerably compared to ***COR*** (almost zero), while C1 and C2 have their partial charges augmented (−0.501 e̅ + 0.430 e̅, respectively).

In the case of pyridinium-N (***P1†*** structure), the changes are less significant, except for the P2 carbon atom bonded to the nitrogen atom, whose charge changes from −0.210 e (in ***COR***) to −0.016 e (in ***P1†***). The hydrogen atom (H1) protonates the nitrogen atom in position P1 and maintains an atomic charge of +0.355 e̅, whose charge goes from −0.210 e̅ (in ***COR***) to −0.016 e̅ (in ***P1†***). In the structures with Y-carbon doped with N, the changes in the charges on the carbon atoms are also less significant than with pyridinic-N in the coronene structure. However, it is worth noticing an important difference: while both pyridinic and pyridinium nitrogens have a negative charge (−0.700 e̅ and −0.242 e̅, respectively), in the graphitic structure, the nitrogen becomes positive in I1 and C1 (+0.307 e̅ and +0.329 e̅, respectively).

### 3.5 Molecular Electrostatic Potential (MEP) Analysis

Molecular electrostatic potential (MEP) maps provide information about the electronic density and help identify areas for electrophilic attack, nucleophilic reactions, and hydrogen bonding interactions. Evaluating electrostatic potential maps can also help us understand the redistribution of electron density in the analyzed systems. The contour map provides a simple way to predict how substituents’ presence affects coronene’s electron distribution. The hot colors over the nuclei (yellow and red) are related to the electron-rich areas, which are more prone to electrophilic substitutions and proton fixation. The cold colors (green and blue) represent electron-deficient portions responsible for nucleophilic attacks and proton repulsion.

**Figure 3:**
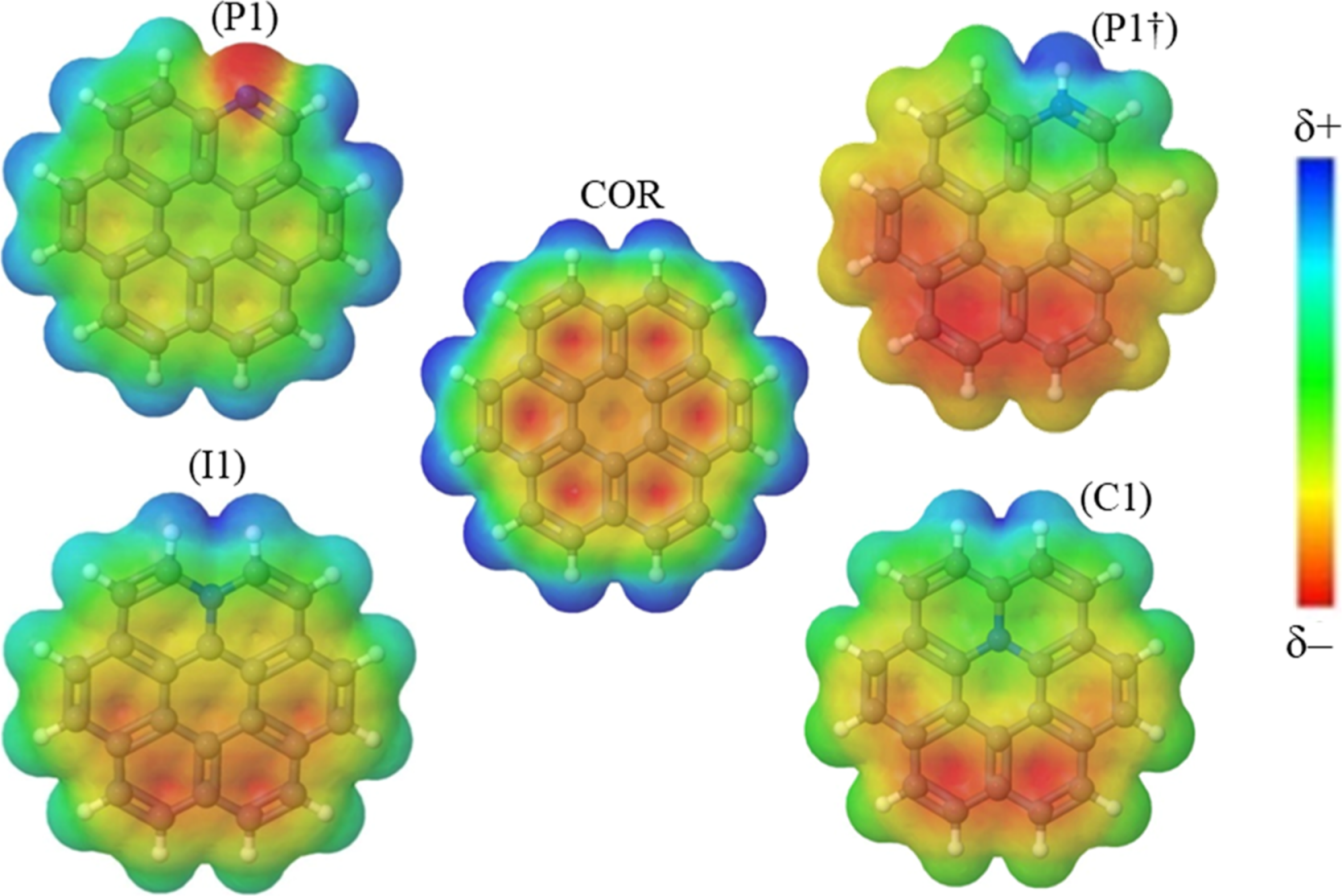
Molecular electrostatic potential maps: ***COR*** and N-COR in ***P1***, ***P†***, ***I1***, and ***C1***, respectively.

The molecular electrostatic potential (MEP) diagram of coronene (Figure 3) reveals that electron deficiencies (shown in blue) are mainly located at the edges of the molecule, making them more susceptible to nucleophilic attack. Moving toward the center of coronene, the availability of electrons increases, resulting in a color change from blue to green, yellow, and red. The red color in the intermediate region indicates a highly negative potential, possibly due to the delocalization of π-electron systems. However, the present MEP and MEV outcomes from the literature^125,126^ indicate a π hole in the central ring due to the electron density depreciation.^127−130^ This π hole can be a center for the Lewis basis attachment, raising coronene’s interaction possibilities with other molecules.

The doping of pyridinic-N in coronene (***P1***) leads to a concentration of negative potential polarized on the nitrogen atom and a considerable decrease of the negative potential on mainly the peripheral rings (color change to green) compared to coronene’s map. After protonation of the nitrogen atom, the ***P1†*** structure has a significant alteration: the positive potential is entirely concentrated on the proton, H1, and the more significant portion of the negative potential goes to the opposite side of the nitrogen atom, occupying roughly half of the structure. However, a similar behavior is observed in the coronene structures doped with graphitic-N, with a smaller magnitude. Therefore, the presence of nitrogen with graphitic arrangement shifts the negative potential in its opposite direction. The nitrogen addition and previous outcomes^127,129^ seem to eliminate the coronene’s π hole and can add a radicalar character to the N-coronene.

### 3.6 Aromaticity

HOMA (Harmonic Oscillator Model of Aromaticity) is the most straightforward, successful, and widely used aromaticity index.^82,131,132^ It embraces the geometrical aspect of aromaticity in which the benzene bond length is the internal standard of perfect aromaticity. The more different from benzene and the more unequal and alternating the bonds are, the lower the HOMA index will be, denoting the extent of decrease in aromaticity.^133^

Values of the HOMA index (eq 2) equal to 1.00 suggest a perfectly aromatic ring (like-benzene), while, equal to 0.00, a perfectly alternating hypothetical Kekulé cyclohexatriene ring. In this work, the aromaticity of the structures was evaluated according to structural method HOMA (eq 2) and energetic criteria of the HOMO–LUMO gap. The values of HOMA, calculated for each ring of the coronene and N-coronene structures, are organized in Table 2. For ***COR***, the peripheral rings (R1–R6) present HOMA values in the range of 0.79–0.80, whereas the central R7 ring’s value is smaller, equal to 0.69. These values are very close to those of Kumar and co-workers,^133^ who used the M06-2X/cc-pVTZ methodology (0.81 and 0.72 for peripheral and central rings, respectively).

**Table 2:** HOMA Values Were Calculated for the Rings in Coronene and N-Substituted Coronene Structures

ring	R1	R2	R3	R4	R5	R6	R7
***COR***	0.79	0.80	0.79	0.79	0.80	0.79	0.69
***P1***	0.87	0.82	0.80	0.80	0.82	0.80	0.74
***P1†***	0.90	0.74	0.84	0.80	0.82	0.87	0.79
***I1***	0.58	0.85	0.77	0.77	0.85	0.58	0.78
***C1***	0.64	0.89	0.75	0.75	0.89	0.64	0.68

The higher HOMA aromaticity of the outer coronene’s rings is consistent with the average C–C bond lengths of 1.407 Å, closer to benzene (1.390 Å). However, the lower average bond length in the central ring (1.423 Å) may be related to a small HOMA index. Some works indicate that aromatic molecules have average C–C bond lengths of 1.41 Å; aliphatic species have such lengths of 1.50 Å or more, and antiaromatic species fall between the two.^134^ Additionally, the molecular electrostatic potential (MEP) maps calculated for the coronene (see Figure 3) show a more intense distribution of red color on the outer rings than on the central ring. This is also supported by Clar’s theory of sextets,^135−137^ which suggests electron delocalization is spread across the outer rings, with no such delocalization within the central ring in the coronene structure. Therefore, both the MEP map and Clar’s theory support the findings of the HOMA analysis.

In the N-coronene structures, the most significant changes in the HOMA occur in ***I1*** and ***C1***, specifically in R1 (and its symmetric R6), where the pyridinic-N is located. Although the average value of the C–C bond lengths in R1 does not show significant changes in ***I1*** and ***C1*** compared to coronene (1.407 Å against 1.402 and 1.404 Å, respectively), the HOMA value undergoes a significant decrease in this ring when compared to COR (from 0.79 to 0.58 and 0.64, respectively). The molecular electrostatic potential (MEP) maps corroborate the HOMA results in R1 for ***I1*** and ***C1***, showing a significant decrease in the red color (shifting toward green) on R1 and its symmetric R6. The indication from HOMA aligns with the results found in the global reactivity descriptors, which indicate increased reactivity when coronene is doped with graphitic-N. This is supported by the decreased hardness and the increased chemical potential in both ***I1*** and ***C1*** compared with COR. Additionally, the HOMA and MEP results suggest that the increased reactivity of doped graphitic-N coronene resulted from the decreased aromaticity in R1, making it more susceptible to reactions.

There is a good similarity between the HOMA values of N-coronene with pyridinic-N (P1, structure) and those of coronene (***COR***), with slight differences highlighted in R1 and R7 (0.87 against 0.79 and 0.74 against 0.69, respectively). However, the MEP image generated for ***P1*** differs significantly from that of ***COR***. That shows that the density of the negative potential (red color) is concentrated on the nitrogen atom, possibly on the lone pair of the pyridinic nitrogen, which is positioned perpendicular to the π system. However, the high similarity between the values of reactivity descriptors for ***PI*** and ***COR*** (Table 1) suggests that these species present similar stabilities/reactivity, as appointed by the HOMA. Other works indicate that this similarity in the stability/reactivity also occurs in similar systems but with different sizes, such as pyridine and benzene or quinoline and naphthalene.^138,139^

### 3.7 QTAIM Analysis

We further studied the effects of substitution in the coronene’s electronic structure using the QTAIM method. As a first approach, the analysis focused on using critical points of a ring to evaluate the structure and compare it with the well-accepted aromaticity indexes. The influence of heteroatoms on electronic density and critical points was analyzed in previous studies found in the literature.^140,141^ Aromaticity is based on electron delocalization of ring systems. The ring critical points (RCPs) for each structure and its corresponding electron density are shown to evaluate this delocalization. The QTAIM analysis shows that the peripheral rings have a higher electron density than the central ring (ρ = 0.0207 au versus 0.0196 au). This result agrees with the HOMA index, MEP, and Clar’s theory. In the N-substituted structures, the electronic density of each RCP differs depending on the position of the substitution. In ***I1***, the addition of the nitrogen atom changes the electronic density of the rings, especially those opposite to the nitrogen (rings 6 and 7). The density slightly decreases compared to that of COR, which was not observed in HOMA or the electrostatic potential map. Although just like the COR, the outer rings continue to have a higher electron density than the inner ring. The most significant modification in the electronic density is observed for the C1 structure. The upper rings have the lowest ρ (0.0202 au) at the RCP compared to those of the other rings. This behavior was also observed in HOMA and the electrostatic potential map, showing that replacing the nitrogen atom in this position reduces the electronic density in the rings it is part of. The central ring remains with a lower density compared to that of the other rings.

The analysis of ***P1*** RCP, HOMA, and the electrostatic potential map shows that most electron density is distributed over the ring containing the nitrogen (ρ = 0.0230 au), followed by the below ring (ring 5). The remaining outer rings have electron density equal to COR (0.0207 au). Again, the inner ring has the lowest electron density at the RCP, corroborating the HOMA and electrostatic potential map analyses. Such results can be rationalized in the light of Clar’s theory. The theory argues that the smaller the number of disjunct sextets present in an HPA, the greater the loss of thermodynamic stability^141^ (and consequently, aromaticity) of the species.^137,142^ Based on this premise, it can be inferred that the addition of nitrogen in the ***I1*** and ***C1*** positions must cause an interruption in the electronic delocalization of the rings that share the central nitrogen atom, leading to a decrease in the number of π sextets (from three to two), a fact not observed in P1. These results show that nitrogen’s replacement of carbon atoms influences the delocalization of π electrons (consequently aromaticity). Therefore, it is expected that the π interactions (π···π, N–H···π, C–H···π) be altered to some extent.

#### 3.7.1 Adsorption of a Single CO_2_ Molecule

**Figure 4:**
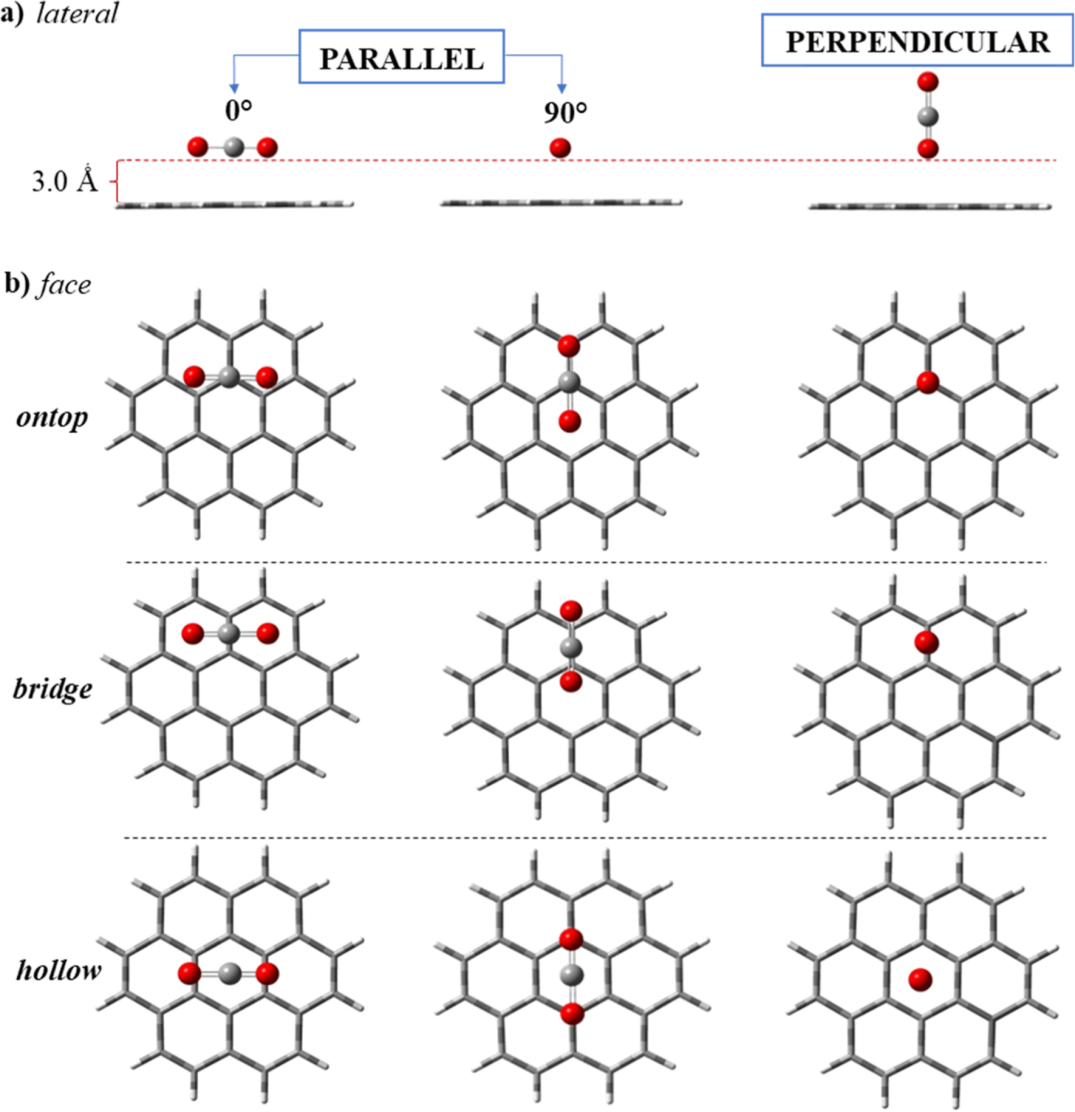
Parallel (0°), parallel (90°), and perpendicular starting orientations for a single CO_2_ molecule approached over the ring, atom, bond, and edge of coronene, represented in the lateral (a) and frontal (or face) (b) views.

Afterward, the coordinates of a single CO_2_ molecule were relaxed on the fixed aromatic plane of the previously optimized molecules, starting from 3.00 Å. The parallel and perpendicular orientations were tested on the hollow, bridge, and ontop sites (Figure 4), generating innumerable starting configurations (Figure S1, Supporting Information). After optimization of the CO_2_ atomic coordinates on aromatic planes of the coronene and coronene-N unit (description detailed in methodology, Figures 1 and S1), it was confirmed that CO_2_ only adsorbs in plane-parallel configurations, consistent with other aromatic models presented in the literature.^31,143^

According to Table 3, the CO_2_ adsorption energies calculated using the CAMB3LYP-D3/6-311G(d,p) methodology present the following order: ***COR*** > ***C1*** > ***P1†*** > ***I1*** > ***P1***, corresponding to values of −3.33 kcal mol^–1^, −3.51 kcal mol^–1^, −3.67 kcal mol^–1^, −3.72 kcal mol^–1^, and −5.92 kcal mol^–1^. These values are in good agreement with theoretical studies employing the C_23_H_12_N···CO_2_ complex (from −3.24 kcal/mol to −3.56 kcal/mol) performed by Xie and collaborators,^144^ using the B3LYP-D3 functional. The exception in the adsorption energy occurs for the CO_2_/***P1*** complex, where the preferential CO_2_ adsorption site occurs laterally to the molecular plane, not over the π system.

**Table 3:** CO_2_ Adsorption Energy (*E*_ads_ in kcal mol^–1^), CO_2_ Adsorption Mode, Smallest X–Y Interatomic Distances in the CO_2_ Adsorption Site (*d*^(X,Y)^ in Å), Largest C=O Bond Length (*d*^(C–O)^ in Å), Total Charge in Adsorbed the CO_2_ Molecule (∑*q*_(CO_2_)_ in e̅), and Charge in the Nitrogen Atom (*q*_(N)_ in e̅), for the CO_2_/COR and CO_2_/N-COR Minimum Energy Complexes

CO_2(ads)_	mode	*E*_ads_	∑*q*_(CO_2_)_	*q*_(N)_	*d*^(C–O)^	*d*(^X^–^Y^)^a^	
***COR***	bridge	–3.33 (−3.03)	–0.001		1.156	3.187^(C–C1)^ 3.362^(O–C1)^	3.187^(C–C2)^ 3.363^(O–C2)^
***P1***	ontop/H bonds	–5.92 (−4.85)	–0.010	–0.696	1.157	2.594^(O–H2)^ 2.714^(C–P1)^	2.528^(O–H12)^ 2.930^(O–P1)^
***P1†***	bridge	–3.67 (−3.13)	+0.012	–0.257	1.157	3.181^(C–C1)^ 3.193^(O–I1)^	3.227^(C–C2)^ 3.174^(O–P1)^
***I1***	bridge	–3.72 (−3.60)	–0.010	+0.341	1.157	3.092^(C–C1)^ 3.163^(O–C1)^	3.100^(O–I1)^ 3.281^(O–C6)^
***C1***	hollow	–3.54 (−3.34)	+0.004	+0.249	1.155	3.148^(C–I2)^ 3.168^(O–C1)^	3.166^(O–C2)^ 3.234^(C–P2)^

a Bond lengths—indicated in Figure 4—and (*E*_ads_) Interaction energy calculated in the Coupled Cluster level (in kcal mol^–1^).

Some aromatic systems may have two possible interaction structures with CO_2_, the “in-plane electrostatic” and the dispersive “π–π stacking”. In most cases, in-plane conformations are more stable than stacking conformations.^145^ Our results show that the ***P1*** substitution was more stable than the other complex and has CO_2_ in the ring plane and that the stacking geometries (***P1†***, ***I1***, and ***C1***) showed a weak binding energy as the nonsubstituted coronene.

Some works have indicated that increasing the size of the π system leads to an increase in the binding energy of small adsorbent molecules, such as CO_2_.^88,144^ Different N-graphene and N-asphaltenes models have shown that the binding energy of CO_2_ is mainly driven by van der Waals force and varies in narrow ranges, without intermolecular charge transferences.^2^ The slight difference among the calculated adsorption energies suggests that the conversion barrier among the different adsorption sites of CO_2_ on coronene and N-coronene must be small.

These findings are corroborated by BOMD calculations (Supporting Information). The simulation results show the CO_2_ molecule moving over the molecular plane during the time evolution of 400 fs, which is expected for systems bound by van der Waals forces. Also, the planarity of CO_2_ over the coronene’s surface is lost. The molecule assumes an angular orientation relative to the coronene’s plane. Due to the low and similar interaction energies, different conformations can be accessed over time according to the thermal energy.

For industrial CO_2_ capture and separation applications, the ideal adsorption energy must balance the capture capacity and ease of material regeneration. On functionalized carbon surfaces, these values range from −5 to −15 kcal/mol.^146^ Very low energies (−3 to −6 kcal/mol, as found in this study) indicate weak adsorption, facilitating regeneration but lower CO_2_ retention capacity. However, the literature shows that combining functionalized structures with polymers increases the adsorption and regeneration capacity of the material.^147−149^

**Figure 5:**
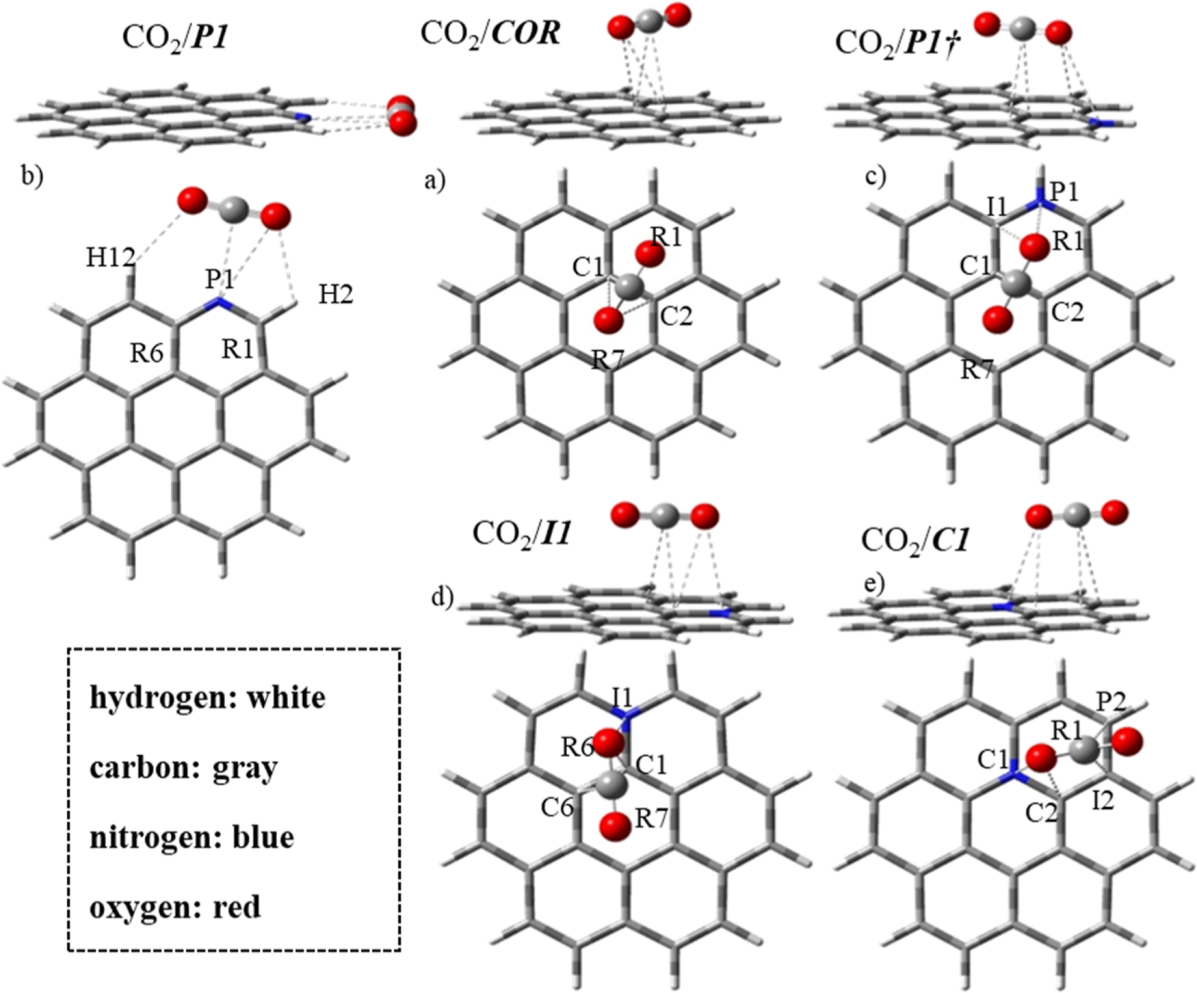
Structures of the complexes (lateral and frontal views) obtained by the interaction between CO_2_ and the coronene and N-coronene units: ***COR*** (a) and ***P1***, ***P1†***, ***I1***, and ***C1*** (b–e, respectively), optimized with the CAM-B3LYP-D3/6-311G(d,p) methodology. The four shortest distances are indicated with sketched lines and labels of the involved atoms and rings.

When the nitrogen atom is in the graphitic arrangement in coronene, the adsorption mode of the CO_2_ molecule is dependent on the position occupied by the nitrogen atom. In the ***C1*** structure, CO_2_ anchors preferentially in the hollow mode in the R1 ring (Figure 5e). One oxygen from CO_2_ approaches the nitrogen (N–O = 3.168 Å) and C2 atom (C–O = 3.166 Å). The other oxygen moves toward the peripheral carbons P2 (C–O = 3.234 Å) and I2 (intermediate carbon 2) (C–O = 3.148 Å). R1 is the preferential adsorption site of CO_2_ in ***C1*** because it has a higher reactivity, indicated by the HOMA index and the MEP calculation. However, the CO_2_ adsorption on ***I1*** occurs preferentially on the R7 ring (Figure 5-d) with shorter C–C1, O–C1, and O–C6 distances: 3.092 Å, 3.100 Å, 3.163 Å, and 3.281 Å, respectively. The positive charge on the nitrogen atom in the ***I1*** structure must be the most essential factor in the CO_2_ molecule adsorption orientation. This positive charge undergoes a slight increase after the adsorption of CO_2_ (Δ*q* = +0.034 e̅), raising the nitrogen acidity. In the opposite behavior, in ***C1***, pyridinic-N receives electron density after CO_2_ adsorption, with its atomic charge going from +0.329 to +0.249 e̅.

**Figure 6:**
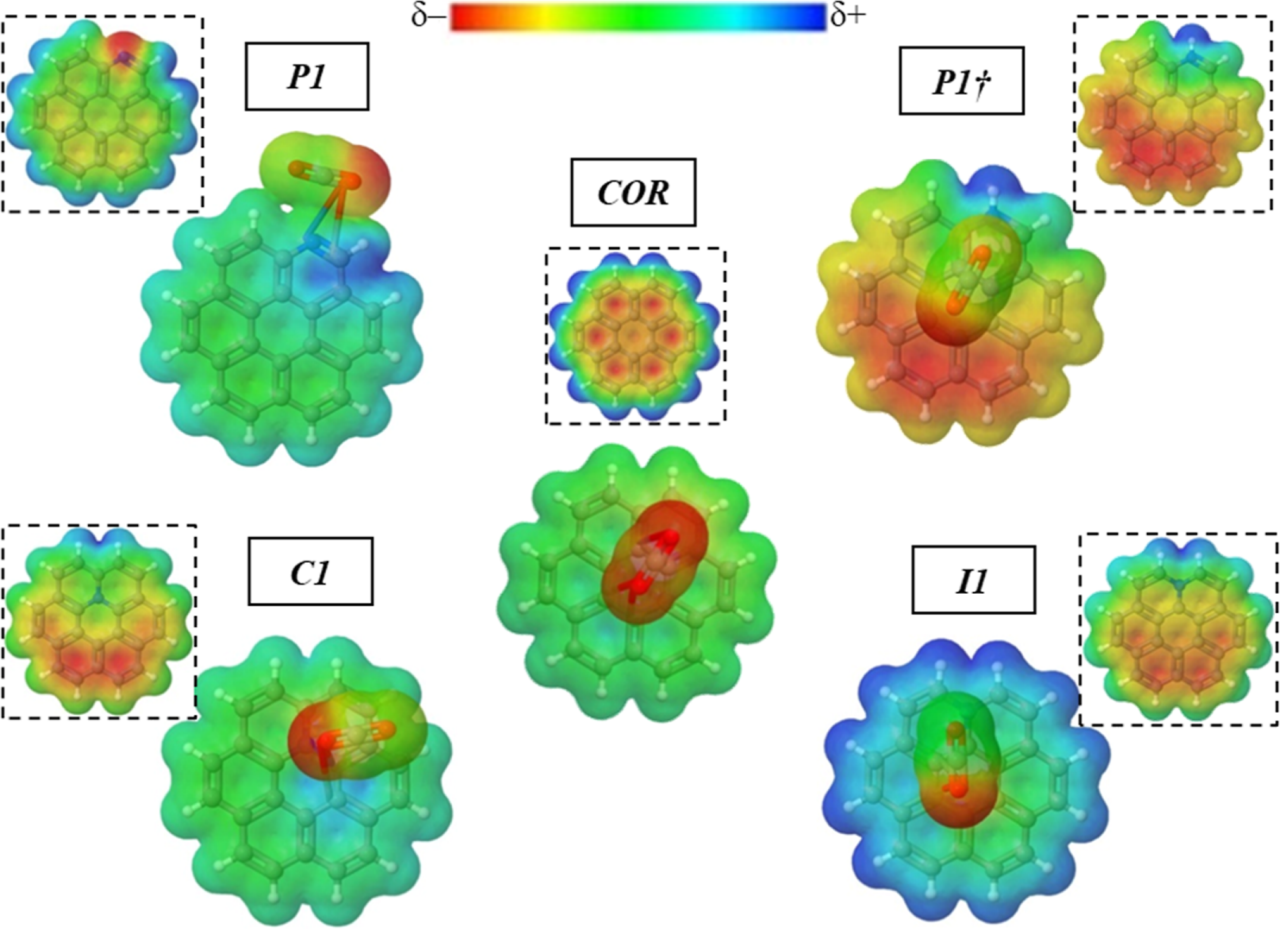
Electrostatic potential map of the ***COR***, ***P1***, ***P1†***, ***I1***, and ***C1*** structures: isolated (in dotted) and complexed with CO_2_.

This different behavior of graphitic-N on the CO_2_ adsorption can be noted by observing the significant difference between the MEP for the CO_2_/***I1*** and the CO_2_/***C1*** complexes (Figure 6). This result suggests that the graphitic-N influence on the adsorption process of CO_2_ on N-coronene will depend on the nitrogen position in the structure.

When the pyridinic N substitution in coronene is performed, the preferential CO_2_ adsorption mode does not involve the π system. Instead, the molecules move toward the nitrogen’s free electron pair (Figure 5b). Welsh and collaborators found a similar result where the CO_2_ molecule adsorbs preferentially in the same plane of the pyrimidine ring, closer to the nitrogen atom.^140^ After the adsorption of CO_2_ on the ***P1*** molecule, there is an increase in the electron density of CO_2_ (total charge = –0.043 e̅). Furthermore, adding CO_2_ reduces the nitrogen charge (−0.700 versus −0.450 e̅), making it less negative. Although it did not interact directly with the aromatic part, the three regions of the structure were slightly affected.

The ***P1*** charge density is modified due to the CO_2_ physiosorption, as seen in Figure 6. Weak polarization interactions between the adsorbate and the surface occur without charge transfer or surface modifications, confirming the physisorption. In the presence of CO_2_, the ESP shows that the complex is polarized due to the CO_2_ presence. The negative charge density is located mainly in the CO_2_ oxygen. At the same time, the coronenes have nearly zero electrostatic potential in almost all structures (II). Only the coronene part interacting directly with the CO_2_ has a positive potential (Figure 6).

The interaction energies refined at the DLPNO-CCSD(T) level with basis set pVDZ are −3.03 and −4.85 kcal mol^–1^ (Table S4, Supporting Information section). These values were close to those found with the DFT level and showed the same trend. The results obtained with both quantum mechanics methods, CAM-B3LYP-D3 and DLPNO-CCSD(T), showed that the CO_2_ interacts with the coronene via nonbonded weak interactions, with the ***P1*** structure presenting the highest interaction energy in both levels. The binding modes and energies of the present agree with the literature results and the fact that CO_2_ and aromatic rings form van der Waals complexes.^31,143,150^

#### 3.7.2 QTAIM, WBI, and NCI Analysis: CO_2_ Adsorption

**Figure 7:**
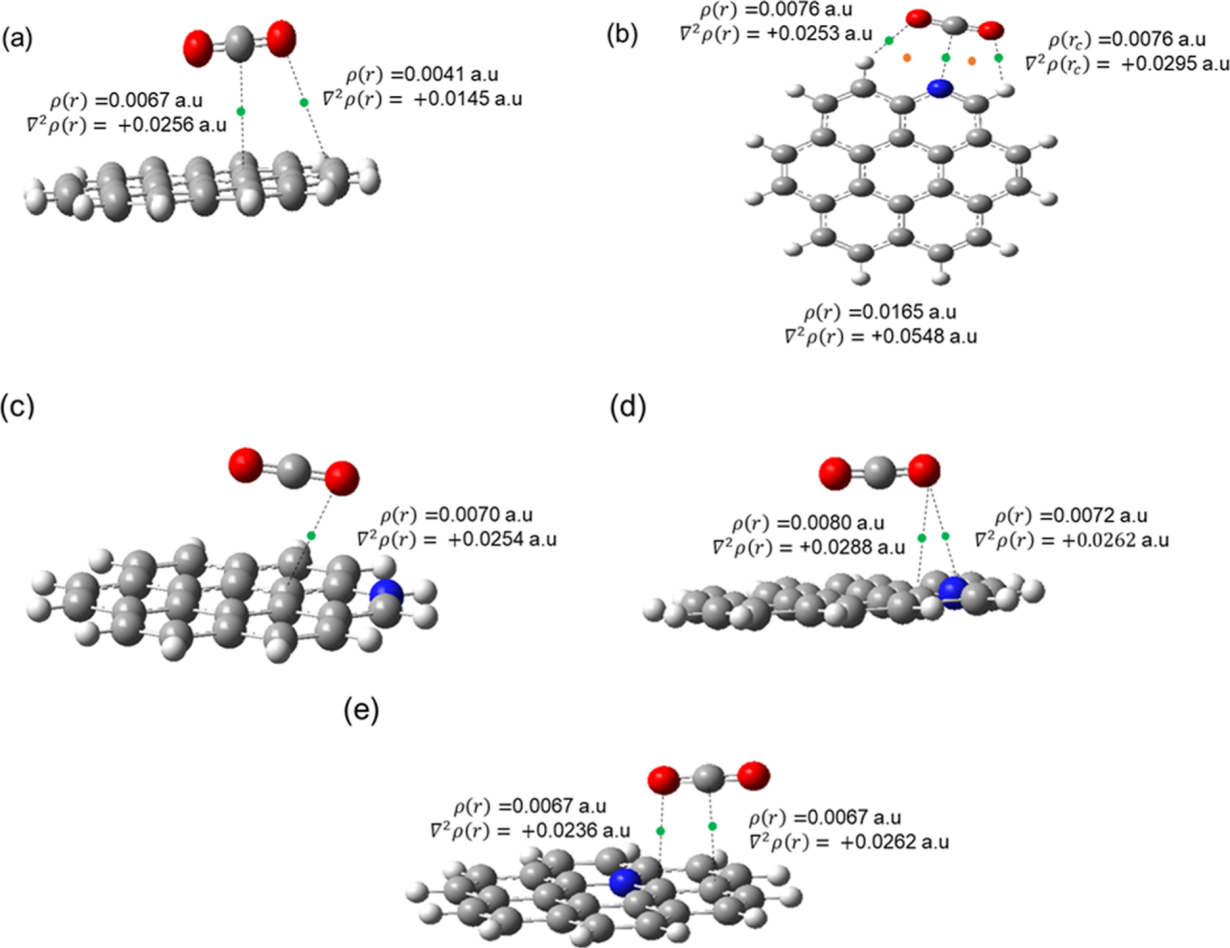
Schematics of BCps(ρ) in ***COR***/CO_2_ complexes. Complexes (a) ***COR***/CO_2_, (b) ***P1***/CO_2_, (c) ***P1†***/CO_2_, (d) ***I1***/CO_2_, and (e) ***C1***/CO_2_. The green spheres represent the binding critical points (BCP), and the orange spheres represent the ring critical points (RCP).

The QTAIM (Quantum Theory of Atoms in Molecules) was used to characterize the noncovalent interactions between the CO_2_/COR complexes, such as vdW and hydrogen bonds. According to the results presented in Figure 7, the electron density between the coronenes and CO_2_ varies with the presence or absence of a heteroatom. All interactions depicted in Figure 7 present the parameters in the range of values corresponding to van der Waals interactions (∼10^–3^ au and positive, respectively).^151^ These results corroborate that CO_2_ forms van der Waals complexes with aromatic compounds such as pyridine, pyrimidine, coronene, etc.^105^Table 4 shows the BCP values found in the complexes.

**Table 4:** Charge Density (ρ in au) and Corresponding Laplacian (∇^2^ ρ in au) in CO_2_/Coronene and CO_2_/N-Coronene Complexes

CO_2(ads)_	BCP	ρ (au)	∇^2^ ρ (au)
***COR***	O···P2	0.0041	+0.0145
	C···C2	0.0067	+0.0256
***P1***	O···H12	0.0076	+0.0253
	C···P1	0.0165	+0.0548
	O···H12	0.0076	+0.0295
***P1†***	C···C2	0.0071	+0.0273
***I1***	O···P1	0.0072	+0.0262
	O···C1	0.0080	+0.0288
***C1***	O···P2	0.0067	+0.0236
	C···C2	0.0067	+0.0262

Four types of noncovalent bonds exist between the CO_2_ molecule and the coronene structures: the C···C, O···C, O···H, and O···N bonds. The ***COR***/CO_2_ complex (Figure 7a) shows two different interactions between CO_2_ and the coronene: the O···C interaction (BCP: ρ = 0.0041 au and ∇^2^ρ = +0.0145 au) and the C···C interaction (BCP: ρ = 0.0067 au and ∇^2^ρ = +0.0256 au).

The ***P1***/ CO_2_ complex (Figure 7b) interacts most with CO_2_ and N-coronene. The QTAIM results show the formation of two different noncovalent bond types: the C···N interaction with BCP: ρ = 0.0165 au and ∇^2^ρ = +0.0548 au and two nonconventional hydrogen bonds O···H BCP: ρ = 0.0076 au and ∇^2^ρ = +0.0253 au and O···H BCP: ρ = 0.0076 au and ∇^2^ρ = +0.0295 au.^152^ In addition, it is possible to observe that the interaction of the CO_2_ molecule with ***P1*** forms two cycles characterized by two RCPs (orange spheres: (ρ(RCP) 0.0055 au and 0.0075 au, respectively). These RCPs reinforce the characterization of two nonconventional hydrogen bonds between ***P1*** and CO_2_. Despite the highest interaction energy calculated for this structure, this arrangement may have a low probability of being observed in real asphaltenes. Due to the sizable aromatic part in asphaltenes, in conjunction with the steric hindrance of the side chains, most CO_2_ interactions with asphaltenes are expected to follow the modes proposed in Figure 7a,c,d,f,e. However, the nonconventional hydrogen bond formation justifies the in-plane structures of CO_2_ with small N-aromatic complexes.^152^

The ***P1†***/CO_2_ complex (Figure 7c) has a single noncovalent interaction between the structures: the O^···^C bond with BCP having ρ = 0.0070 au and ∇^2^ρ = +0.0254 au. This complex is the only one involving N-substituted structures where CO_2_ interacts with the surface through only one atom. In the other structures, the CO_2_ molecule also interacts through the carbon atom with carbons in the neighborhood of the heteroatom. In the ***I1***/CO_2_ complex (Figure 7d), the CO_2_ molecule interacts with the coronene through only one oxygen atom from CO_2_. However, there are two different types of interaction between CO_2_ and the N-coronene: the O···N interaction with BCP: ρ = 0.0073 au and ∇^2^ρ = +0.0262 au, and the O···C BCP interaction: ρ = 0.0081 au and ∇^2^ρ = +0.0288 au. In the ***C1***/CO_2_ complex (Figure 7e), unlike the other nitrogenous structures, CO_2_ does not interact with the nitrogen atom and there are two different types of interaction of smaller magnitude than those found in the other complexes, the O^···^C with BCP having ρ = 0.0067 au and ∇^2^ρ = +0.0236 au and the C^···^C BCP interaction with ρ = 0.0067 au and ∇^2^ρ = +0.0262 au.

Wiberg binding indices (WBIs) were used to evaluate the bond orders in the ***COR***, ***P1***, and ***P1†***/CO_2_ complexes to deeply understand the CO_2_ coronene interaction. The closer the atoms occur to each other for the same type of chemical bond, the stronger the Wiberg binding index.^153^ The WBI results in the ***COR***/CO_2_ complex showed that the C···C interaction is equal to 0.0021, which indicates a weak interaction.^154^ In this complex, the WBI of the O···C interaction of 0.0002 is almost negligible. The P1/CO2 complex results show WBIs for the O···H interactions of 0.0013 and 0.0022, respectively. The C···N presented in the CO_2_ interaction the highest index, 0.0210. In all cases, the values describe weak noncovalent interactions. The H atom presence and the cationic character of the ***P1†***/CO_2_ complex change the WBI to 0.0004, which is also considered a negligible CO_2_ interaction. All values found are lower than 0.5, which indicates weak interactions (in the present case, nonbonded interactions), and therefore, these structures may serve as adsorbents for CO_2_ by physiosorption.^80,153,154^

**Figure 8:**
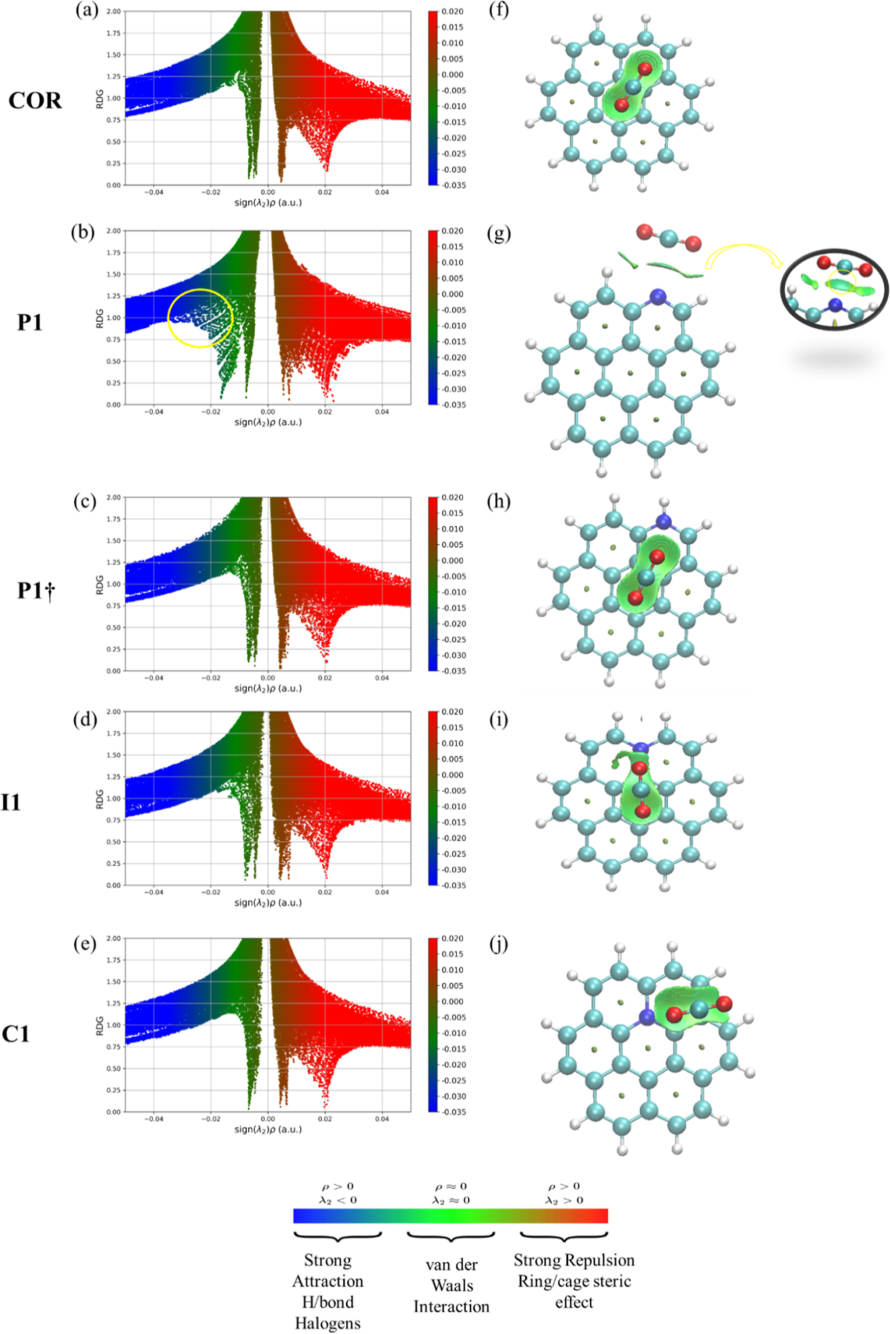
RDG scatter plots (left) and noncovalent interaction (NCI) plots (right) of coronene (a,f) and N-coronene: *P1* (b,g), ***P1†*** (c,h), ***I1*** (d,i), and ***C1*** (e,j). The isosurfaces are colored according to the values of sign()r (au), from 0.03 to 0.02 au. Color on line: blue represents strong attractive interactions, green indicates van der Waals interactions, and red indicates repulsive/steric interactions.

The noncovalent nature of the interaction between the CO_2_ molecule and the coronenes, already seen by the QTAIM analysis, was also confirmed by the NCI analysis (Figure 8 through the 2D (a–e) and 3D (f–j) graphs). The reduced density gradient (RDG) plots show different colors representing the reduced density gradient value change according to the sign of (λ_2_)ρ (where λ_2_ is the second eigenvalue of the Hessian matrix, and ρ is the electron density). Different graph regions discern three types of noncovalent interactions (i.e., the hydrogen bond, van der Waals, and the repulsive steric effect). Figure 8 shows a green peak in the low-density region ((*s*(signλ_2_)ρ) = −0.01 au) as well as the green region between the structures in the 3D graph. These results indicate that the coronenes and CO_2_ interactions are mainly van der Waals. Also, is present a red peak in the region of (λ_2_)ρ = 0.02 au indicating a steric repulsion caused by the proximity between the molecules (Figure 8a,b), which is expected. The 2D graphs show more expressive green density regions for the substituted structures (Figure 8b–d) than the nonsubstituted (Figure 8a). These results agree with the interaction energies calculated for all structures. The substituted ones are more prone to interact with the CO_2_ molecule than the nonsubstituted. The analysis is very similar in all structures, except for ***P1***, where it is possible to observe a slight change in the blue region of the graph associated with the formation of nonconventional hydrogen bonds. This result corroborates the QTAIM analysis, which pointed out two nonconventional hydrogen bonds between CO_2_ and the ***P1*** (Figure 8b, yellow circle). The 3D graph (Figure 8g) also shows a light blue color in the middle of the green surface related to the interaction of CO_2_ oxygens with the hydrogen atoms for ***P1***. Still, according to the 3D graph (Figure 8f–j), it is possible to observe a slight difference in the density distribution due to the change in position during the substitution. The green region between the structures indicates that the essential factor for these interactions is van der Waals interactions, regardless of whether substitution occurs or not.

Noncovalent interactions play a crucial role in CO_2_ adsorption by stabilizing all complexes. These interactions are essential to maintain adsorbed CO_2_ in the structure. Van der Waals interactions contributed to the majority of the total adsorption energy. Although van der Waals interactions are relatively weak individually, together, they can provide a significant contribution to the adsorption energy. The presence of a heteroatom may influence the selectivity of the adsorbent toward CO_2_. Tuning the van der Waals interactions and other attractive interactions due to heteroatoms can lead to the more efficient and selective adsorption of CO_2._

The results showed that the nonconventional hydrogen bond between the CO_2_ molecule and the ***P1*** structure was relevant to the adsorption. Forming these unconventional hydrogen bonds in the pyridine substitution favored complex stabilization. The hydrogen bond formation and the CO_2_ in-plane orientation are the main differences between the results found in the ***P1*** and ***P1†*** positions. In both structures, substitution occurred at the edge of the ring; however, the ***P1†*** structure does not form hydrogen bonds with the CO_2_ molecule. In the P1 case, the hydrogen bonds helped to orient the CO_2_ molecule to the edge, leading to hydrogen bond formation at the plane. This result demonstrates the importance of the heteroatom position and reinforces its influence on adsorption.

**Figure 9:**
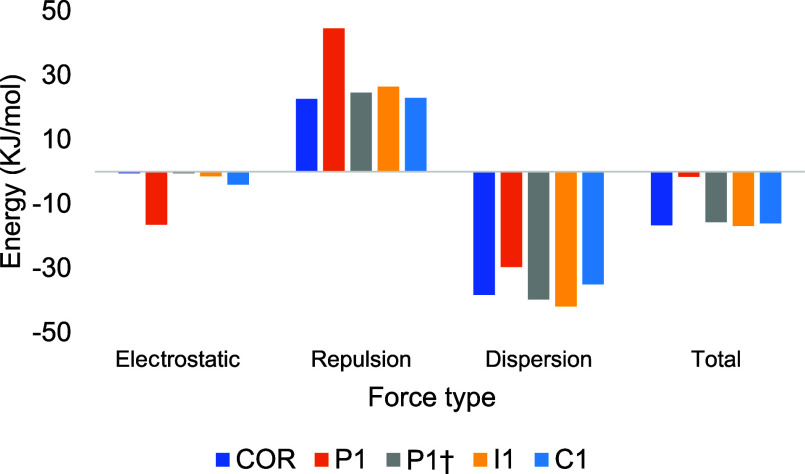
Energy decomposition analysis (EDA–FF) of interaction calculated by the AMBER force field (in percentage) in the MON/CO_2_ complexes.

To determine the contributions to the binding energy (dispersion, electrostatic, and Pauli repulsion) between coronenes and CO_2_, an energy decomposition evaluation based on the AMBER force field (EDA–FF) was performed.^85,86^ These results are shown in Figure 9.

The literature shows that the interaction of CO_2_ with aromatic structures involves a competition between electrostatic and dispersion interactions.^145^ Since aromatic systems have a π cloud, they enable dispersion interactions with gases. However, this competition was not observed because the dispersion is the major contribution and the electrostatic component is almost negligible. In all complexes, dispersion and electrostatic energy had negative values, indicating attractive components of the intermolecular interaction. In complex ***P1***, the largest contribution came from repulsion energy. The ***P1*** complex is stable because the sum of the electrostatic and dispersion forces surpasses the repulsive contribution. The electrostatic component in ***P1*** is greater than that was obtained for the other conformations, reinforcing the formation of a hydrogen bond.

The results of this study can be extended to other PAHs because their basic structure shares similar electronic and interaction characteristics with those of CO_2_. However, when functionalization occurs, it is necessary to analyze the position. The study showed that pyridine nitrogen could lead to the formation of nonconventional hydrogen bonds, while the other structures favored the interaction of CO_2_ with the PAH π system. In a possible substitution with pyrrole nitrogen, the presence of electron pairs available for electrostatic interactions, as in pyridine, can increase the level of CO_2_ adsorption.

## 4 Conclusions

In the present study, the influence of a nitrogen dopant on the adsorptive capability of molecular coronene, corresponding to CO_2_ adsorption, was investigated at DFT-D3 and DLPNO-CCSD(T) levels. Geometrical parameters, charge distributions, intra- and intermolecular charge transferences, Kohn–Sham frontier molecular orbitals, global reactivity descriptors, electrostatic potential maps, aromaticity, and topological parameters were evaluated for isolated coronene and N-coronene and for structures containing CO_2_.

The results, in the absence of CO_2_, showed that the COR and P1 structures present high similarity in the energy levels of the frontier orbitals (HOMO and LUMO). Thus, these two species present high similarity in all reactivity descriptors employed. However, when the pyridine-N is protonated, the cationic structure N-coronene (+) (***P1†***) presents a greater reactivity of all the calculated structures. The structures of ***I1*** and ***C1*** have lower hardness and higher chemical potential, reducing their greater tendency for charge transfer. The HOMA analysis indicates a high similarity in aromaticity between ***COR*** and ***P1***.

However, when the interaction with CO_2_ is evaluated, nitrogen doping generally does not significantly alter the adsorption energy, except for pyridine complex ***P1***. The CO_2_ molecule adsorbs parallel to the coronene, regardless of the initial structure of the optimization procedure, which considered the CO_2_ parallel or perpendicular to the surface. All calculated adsorption energies are low (−5 < Δ*E* < −3 kcal mol^–1^), indicating the formation of van der Waals complexes and suggesting a physisorption process. For this reason, interconversion between the complex structures should occur. Also, a physiosorption process indicates that the pressure may play an important role in the process and that the adsorbent can be regenerated.

In the case of the ***P1***/CO_2_ complex, the in-plane electrostatic interaction was the differential effect that stabilized the structure due to the formation of the nonconventional hydrogen bond with CO_2_. The QTAIM, NCI, and WBI analyses confirmed the formation of hydrogen bonds. The EDA–FF analysis showed that dispersion significantly contributes to the COR and N-COR/CO_2_ interactions. The EDA-FF also confirmed the nonconventional hydrogen bond in the ***P1*** complex.

The study results provide guiding principles to produce new adsorbents with improved properties and selectivity for CO_2_, such as carefully choosing the substitution position. The results can inspire combined strategies to create active surfaces for CO_2_ adsorption as well as inhibitors for asphaltene aggregation. For example, acid inhibitors that will react with the nitrogen or molecules that can eliminate the free radical character of the substituted coronenes. CO_2_ capture systems can also be based on the outcomes of the present study. Since the interactions addressed are weak, pressure should be an important factor in projecting adsorbers based on the present systems.

Subsequent studies will evaluate the synergistic effect of two types of nitrogen doping and the N-pyrrolic dopation. Also, the dimerization of coronene will be studied to describe asphaltene’s precipitation and the substituent effect on this process.

## Supporting Information Available

BOMD simulations showing the interaction between CO_2_ and N-doped coronene in the graphitic arrangement (MP4)
BOMD simulations showing the interaction between CO_2_ and N-doped coronene in the pyridinic arrangement (MP4)
All bond lengths present in the optimized isolated structures; MKS atomic charge on the atoms in the optimized isolated structures; ChelpG, MSK, NBO, and Hirshfeld atomic charge on the atoms in the optimized isolated structures; CO_2_ adsorption mode for all structures, interaction energy in Coupled Cluster, DFT method, and T1 diagnostic; and MKS atomic charge on the atoms in the CO_2_ complexes structures (PDF)
