# Supplementary material for: Study of the Electronic Structure of Coronene Doped with Nitrogen Atoms and Its Effect on CO2 Capture
Source: ACS Omega. 2025 Apr 16;10(16):16559–78. doi: 10.1021/acsomega.4c11531 (PMC12044568; doi:10.1021/acsomega.4c11531)
Supplement: Supplementary file 3 — ao4c11531_si_003.pdf [file ao4c11531_si_003.pdf]

Supporting Information

Study of the electronic structure of coronene doped with nitrogen atoms and its effect on CO_2 capture

Kelly F. P. Laeber[a‡], Letícia M. Prates[b‡], Leonardo Baptista[c‡], Maurício T. M. Cruz[a‡]*

[a]Departamento de Química Geral e Inorgânica, Universidade do Estado do Rio de Janeiro, Rua São Francisco Xavier, 524, Maracanã, Rio de Janeiro – RJ,Brasil, CEP 20550-900;

[b]Centro de Tecnologia Mineral Avenida Pedro Calmon, 900 - Cidade Universitária - Rio de Janeiro–RJ, Brasil, CEP 21941-908;

[c]Departamento de Química e Ambiental, Universidade do Estado do Rio de Janeiro, Faculdade de Tecnologia, Av. Dr. Omar Dibo Calixto Afrange, s/n - Polo Industrial, Resende – RJ, Brasil. CEP 27537-000

Table of Contents

Table S1. All bond lengths present in the optimized isolated structures of coronene and N–coronene, considering their highest point group. …………..S4

Table S2. MKS atomic charge on the atoms of coronene and N–coronene. …………..S5

Table S3. Chelpg, MSK, NBO, and Hirshfeld atomic charges on the atoms of coronene. …………..S6

Table S4. Chelpg, MSK, NBO and Hirshfeld atomic charges on the atoms of N–coronene (N-pyridinic). …………..S7

Table S5. Chelpg, MSK, NBO and Hirshfeld atomic charges on the atoms of N–coronene (N-quaternary). …………..S8

Scheme S1. MSK atomic charge distribution (in ē): on the coronene (COR) e N–coronene (P1, P1†, I1 and C1) units (in rows); on the Peripheral, Intermediate and Central regions (in collumns) and in hydrogen atoms (in collumn). …………..S9

Scheme S2. MSK atomic charge variation distribution (in ē) in CO_2/coronene complexes. Total for the coronene (*COR*) e N–coronene (*P1*, *P1†*, *I1*, and *C1*)/CO_2 complexes depicted in rows, while separated by regions (peripheral, intermediate, and central) and in hydrogen atoms, depicted in columns. …………..S9

Table S6. MKS atomic charge on the atoms of the coronene/CO_2 and N–coronene/CO_2 complexes in configurations of lowest energy. …………..S10

Table S7. CO2 adsorption mode, interaction energy (Eint, in kcal mol-1), and CO_2–aromatic plane ($D_{CO2/plane}$) and CO_2–N ($D_{CO2\text{-}N}$) distances (in Å), in the CO_2/coronene and CO_2/N-coronene complexes optimized at the DFT level. …………..S11

Table S8. Energy (in Hartree) and T1 diagnostic of the coronene and N–coronene structures. …………..S12

Table S9. Interaction energy (Eint in kcal mol^{-1}) at Coupled Cluster level. ………..S12

Figure S1. All CO_2 adsorption sites tested on the coronene (COR) and N–coronene (P1, P1†,I1 e C1), considering the molecular symmetry. ……….....S13

Figure S2. Geometries for all CO_2 configurations obtained on the coronene (COR) and N–coronene (P1, P1†,I1 e C1) species. ………....S14

Scheme S3. C–C and C–N bond lengths (in Å) in the different regions in coronene and N-coronene (pyridinic–N): *COR* versus *P1* (a), *COR* versus *P1†* (b) and *P1* versus *P1†* (c). ………....S15

Scheme S4. C–C and C–N bond lengths (in Å) in the different regions in coronene and N-coronene (Schemeitic–N): *COR* versus *I1* (a), *COR* versus *C1* (b) and *I1* versus *C1* (c). ………....S16

Table S1. All bond lengths (in Å) present in the optimized isolated structures of coronene and N–coronene, considering their highest point group.

Structure	***COR***	***P1***	***P1†***	***I1***	***C1***
Point group	D_{6h}	C_s	C_s	C_{2v}	C_{2v}
Bond length			Peripheral		
P1–P2		(1.301)	(1.332)	1.339	1.359
P3–P4		1.363	1.357	1.380	1.367
P5–P6	1.361	1.362	1.367	1.361	1.356
P7–P8		1.362	1.361	P5–P6	P5–P6
P9–P10		1.363	1.363	P3–P4	P3–P4
P11–P12		1.363	1.366	P1–P2	P1–P2
			Peripheral–Intermediary		
P1–I1		(1.366)	1.375	(1.388)	1.401
P2–I2		1.424	1.389	1.445	1.437
P3–I2		1.420	1.426	1.394	1.409
P4–I3		1.422	1.428	1.409	1.406
P5–I3		1.421	1.413	1.424	1.426
P6–I4	1.420	1.421	1.417	1.418	1.424
P7–I4		1.421	1.421	P6–I4	P6–I4
P8–I5		1.421	1.421	P5–I3	P5–I3
P9–I5		1.421	1.420	P4–I3	P4–I3
P10–I6		1.421	1.419	P3–I2	P3–I2
P11–I6		1.424	1.419	P2–I2	P2–I2
P12–I1		1.420	1.408	P1–I1	P1–I1
			Intermediary–Central		
I1–C1		1.400	1.401	1.408	(1.424)
I2–C2		1.403	1.418	1.416	1.399
I3–C3	1.408	1.405	1.410	1.416	1.415
I4–C4		1.406	1.406	1.414	1.396
I5–C5		1.406	1.407	I3–C3	I3–C3
I6–C6		1.405	1.407	I2–C2	I2–C2
			Central		
C1–C2		1.414	1.415	1.407	(1.405)
C2–C3		1.418	1.412	1.422	1.411
C3–C4	1.423	1.422	1.420	1.419	1.424
C4–C5		1.423	1.419	C3–C4	C3–C4
C5–C6		1.421	1.418	C2–C3	C2–C3
C6–C1		1.418	1.417	C1–C2	C1–C2
			Hydrogen–Peripheral		
H1–P1			1.013	1.081	1.083
H2–P2		1.088	1.082	1.082	1.082
H3–P3		1.084	1.083	1.084	1.084
H4–P4		1.084	1.083	1.083	1.083
H5–P5		1.088	1.083	1.084	1.084
H6–P6	1.084	1.088	1.084	1.084	1.084
H7–P7		1.088	1.083	H1–P1	H1–P1
H8–P8		1.088	1.083	H2–P2	H2–P2
H9–P9		1.088	1.083	H3–P3	H3–P3
H10–P10		1.088	1.083	H4–P4	H4–P4
H11–P11		1.088	1.083	H5–P5	H5–P5
H12–P12		1.082	1.084	H6–P6	H6–P6

( ) N–C bond length. [ ] Equivalence of bond length caused by symmetry.

Table S2. MKS atomic charge on the atoms of coronene and N–coronene optimized isolate.

Atom notation	***COR***	***P1***	***P1†***	***I1***	***C1***
P1	–0.210	**–0.700**	**–0.242**	–0.238	–0.162
P2	–0.225	+0.355	–0.016	–0.291	–0.325
P3	–0.220	–0.137	–0.229	–0.241	–0.253
P4	–0.220	–0.233	–0.160	–0.270	–0.259
P5	–0.225	–0.249	–0.235	–0.268	–0.275
P6	–0.210	–0.185	–0.127	–0.190	–0.204
P7	–0.226	–0.219	–0.189	–0.190	–0.204
P8	–0.187	–0.220	–0.186	–0.268	–0.275
P9	–0.200	–0.260	–0.173	–0.270	–0.259
P10	–0.200	–0.122	–0.164	–0.241	–0.253
P11	–0.187	–0.109	–0.052	–0.291	–0.325
P12	–0.226	–0.368	–0.328	–0.238	–0.162
I1	+0.127	+0.723	+0.234	**+0.307**	–0.034
I2	+0.135	–0.272	+0.054	+0.170	+0.234
I3	+0.135	+0.202	+0.172	+0.196	+0.218
I4	+0.127	+0.104	+0.110	+0.101	+0.132
I5	+0.088	+0.169	+0.138	+0.196	+0.218
I6	+0.088	–0.062	+0.024	+0.170	+0.234
C1	–0.004	–0.501	–0.050	–0.019	**+0.329**
C2	–0.003	+0.430	+0.119	–0.017	–0.116
C3	–0.003	–0.186	–0.048	–0.032	–0.030
C4	–0.004	+0.092	+0.024	+0.031	+0.025
C5	+0.014	–0.099	–0.024	–0.032	–0.030
C6	+0.014	+0.238	+0.066	–0.017	–0.116
H1	+0.152		+0.355	+0.178	+0.157
H2	+0.154	+0.067	+0.184	+0.179	+0.163
H3	+0.155	+0.146	+0.187	+0.156	+0.160
H4	+0.155	+0.152	+0.175	+0.157	+0.156
H5	+0.154	+0.160	+0.176	+0.155	+0.161
H6	+0.152	+0.152	+0.166	+0.145	+0.149
H7	+0.153	+0.155	+0.172	+0.145	+0.149
H8	+0.147	+0.155	+0.171	+0.155	+0.161
H9	+0.148	+0.158	+0.168	+0.157	+0.156
H10	+0.148	+0.139	+0.166	+0.156	+0.160
H11	+0.147	+0.146	+0.165	+0.179	+0.163
H12	+0.153	+0.178	+0.197	+0.178	+0.157

In bold: charge on nitrogen atom.

Table S3. Chelpg, MSK, NBO and Hirshfeld atomic charge on the atoms of coronene and N–coronene.

Atom	***COR***			
	Chelpg	MSK	NBO	Hirsh
P1	–0.229	–0.210	–0.177	–0.047
P2	–0.178	–0.225	–0.177	–0.047
P3	–0.182	–0.220	–0.177	–0.005
P4	–0.182	–0.220	–0.177	–0.047
P5	–0.178	–0.225	–0.177	–0.047
P6	–0.229	–0.210	–0.177	–0.047
P7	–0.229	–0.226	–0.177	–0.047
P8	–0.178	–0.187	–0.177	–0.047
P9	–0.182	–0.200	–0.177	–0.047
P10	–0.182	–0.200	–0.177	–0.047
P11	–0.178	–0.187	–0.177	–0.047
P12	–0.229	–0.226	–0.177	–0.047
I1	+0.200	+0.127	–0.049	–0.005
I2	+0.149	+0.135	–0.049	+0.000
I3	+0.149	+0.135	–0.049	–0.005
I4	+0.204	+0.127	–0.049	–0.005
I5	+0.149	–0.088	–0.049	–0.005
I6	+0.149	–0.088	–0.049	–0.005
C1	–0.033	–0.004	–0.008	+0.000
C2	–0.009	–0.003	–0.008	+0.050
C3	–0.009	–0.003	–0.008	+0.000
C4	–0.033	–0.004	–0.008	+0.000
C5	–0.009	+0.014	–0.008	+0.000
C6	–0.009	+0.014	–0.008	+0.000
H1	+0.130	+0.152	+0.206	+0.050
H2	+0.119	+0.154	+0.206	+0.050
H3	+0.114	+0.155	+0.206	+0.050
H4	+0.114	+0.155	+0.206	+0.050
H5	+0.119	+0.154	+0.206	+0.050
H6	+0.130	+0.152	+0.206	+0.050
H7	+0.130	+0.153	+0.206	+0.050
H8	+0.119	+0.147	+0.206	+0.050
H9	+0.114	+0.148	+0.206	+0.050
H10	+0.114	+0.148	+0.206	–0.047
H11	+0.119	+0.147	+0.206	+0.050
H12	+0.130	+0.153	+0.206	+0.050

Table S4. Atomic charge on the atoms of N–coronene optimized isolate in different models (Nitrogen pyridinic).

Atom	***P1***				***P1†***			
	Chelpg	MSK	NBO	Hirsh	Chelpg	MSK	NBO	Hirsh
P1	**–0.620**	**–0.700**	**–0.452**	**–0.195**	**–0,344**	**–0.242**	**–0,450**	**–0,346**
P2	+0.281	+0.355	+0.116	+0.039	+0,048	–0.016	+0,162	+0,106
P3	–0.150	–0.137	–0.170	–0.042	-0,174	–0.229	–0,164	–0,074
P4	–0.193	–0.233	–0.180	–0.045	-0,154	–0.160	–0,151	–0,065
P5	–0.202	–0.249	–0.181	–0.046	-0,197	–0.235	–0,181	–0,077
P6	–0.184	–0.185	–0.167	–0.042	-0,143	–0.127	–0,120	–0,055
P7	–0.213	–0.219	–0.176	–0.046	-0,169	–0.189	–0,150	–0,070
P8	–0.200	–0.220	–0.175	–0.045	-0,174	–0.186	–0,163	–0,073
P9	–0.249	–0.260	–0.178	–0.046	-0,168	–0.173	–0,150	–0,068
P10	–0.145	–0.122	–0.173	–0.044	-0,154	–0.164	–0,159	–0,072
P11	–0.203	–0.109	–0.175	–0.044	-0,071	–0.052	–0,110	–0,052
P12	–0.202	–0.368	–0.181	–0.042	-0,295	–0.328	–0,234	–0,090
I1	+0.494	+0.723	+0.179	+0.052	+0,309	+0.234	+0,205	+0,126
I2	–0.082	–0.272	–0.098	–0.013	+0,063	+0.054	–0,108	–0,003
I3	+0.187	+0.202	–0.037	+0.000	+0,213	+0.172	–0,003	+0,017
I4	+0.175	+0.104	–0.05	–0.005	+0,161	+0.110	–0,045	+0,003
I5	+0.205	+0.169	–0.046	–0.004	+0,196	+0.138	–0,022	+0,009
I6	+0.120	–0.062	–0.049	–0.005	+0,106	+0.024	–0,047	+0,002
C1	–0.223	–0.501	–0.046	–0.007	-0,081	–0.050	–0,034	+0,011
C2	+0.191	+0.430	+0.015	+0.006	+0,133	+0.119	+0,064	+0,034
C3	–0.075	–0.186	–0.017	+0.000	-0,078	–0.048	–0,033	+0,003
C4	+0.002	+0.092	–0.006	+0.001	+0,010	+0.024	+0,001	+0,007
C5	–0.054	–0.099	–0.009	+0.000	-0,047	–0.024	–0,013	+0,004
C6	+0.055	+0.238	–0.009	–0.002	+0,039	+0.066	–0,001	+0,007
H1					+0,367	+0.355	+0,428	+0,368
H2	+0.048	+0.067	+0.182	+0.054	+0,156	+0.184	+0,230	+0,154
H3	+0.115	+0.146	+0.209	+0.054	+0,141	+0.187	+0,225	+0,119
H4	+0.120	+0.152	+0.208	+0.052	+0,145	+0.175	+0,228	+0,122
H5	+0.124	+0.160	+0.208	+0.052	+0,144	+0.176	+0,225	+0,118
H6	+0.120	+0.155	+0.207	+0.052	+0,148	+0.166	+0,224	+0,121
H7	+0.129	+0.155	+0.207	+0.051	+0,141	+0.172	+0,223	+0,118
H8	+0.127	+0.155	+0.207	+0.051	+0,141	+0.171	+0,223	+0,117
H9	+0.135	+0.158	+0.207	+0.051	+0,141	+0.168	+0,224	+0,119
H10	+0.115	+0.139	+0.207	+0.052	+0,137	+0.166	+0,223	+0,117
H11	+0.130	+0.146	+0.207	+0.052	+0,142	+0.165	+0,228	+0,125
H12	+0.122	+0.178	+0.220	+0.056	+0,167	+0.197	+0,227	+0,120

In bold: charge on nitrogen atom.

Table S5. Atomic charge on the atoms of N–coronene optimized isolate in different models. (Nitrogen Y-Carbon).

Atom	*I1*				*C1*			
	MSK	Chelpg	NBO	Hirsh	Chelpg	MSK	NBO	Hirsh
P1	–0.165	–0.238	+0.036	+0.048	–0.238	–0.162	–0.191	–0.074
P2	–0.245	–0.291	–0.234	–0.064	–0.184	–0.325	–0.226	–0.074
P3	–0.225	–0.241	–0.191	–0.058	–0.189	–0.253	–0.175	–0.049
P4	–0.218	–0.270	–0.201	–0.062	–0.207	–0.259	–0.194	–0.057
P5	–0.193	–0.268	–0.193	–0.055	–0.179	–0.275	–0.181	–0.005
P6	–0.217	–0.190	–0.168	–0.047	–0.226	–0.204	–0.173	–0.049
P7	–0.217	–0.190	–0.168	–0.047	–0.226	–0.204	–0.173	–0.012
P8	–0.193	–0.268	–0.193	–0.055	–0.179	–0.275	–0.181	–0.047
P9	–0.218	–0.270	–0.201	–0.062	–0.207	–0.259	–0.194	–0.003
P10	–0.225	–0.241	–0.191	–0.058	–0.189	–0.253	–0.175	–0.005
P11	–0.245	–0.291	–0.234	+0.010	–0.184	–0.325	–0.226	+0.047
P12	–0.165	–0.238	+0.036	+0.010	–0.238	–0.162	–0.191	–0.054
I1	**+0.187**	**+0.307**	**–0.389**	**–0.033**	+0.196	–0.034	+0.123	+0.029
I2	0.191	+0.170	–0.065	–0.018	+0.128	+0.234	–0.082	–0.016
I3	+0.163	+0.196	–0.038	–0.004	+0.151	+0.218	–0.042	–0.007
I4	+0.187	+0.101	–0.07	–0.015	+0.200	+0.132	–0.067	–0.047
I5	+0.163	+0.196	–0.038	–0.004	+0.151	+0.218	–0.042	–0.049
I6	+0.191	+0.170	–0.065	–0.018	+0.128	+0.234	–0.082	–0.057
C1	–0.012	–0.019	+0.178	+0.042	**–0.032**	**+0.329**	**–0.381**	**–0.049**
C2	–0.027	–0.017	–0.025	–0.005	+0.046	–0.116	+0.228	–0.016
C3	+0.005	–0.032	–0.012	–0.001	–0.010	–0.030	–0.046	–0.029
C4	–0.050	+0.031	–0.008	–0.002	–0.039	+0.025	+0.000	+0.061
C5	+0.005	–0.032	–0.012	–0.001	–0.010	–0.030	–0.046	–0.007
C6	–0.027	–0.017	–0.025	–0.005	+0.046	–0.116	+0.228	+0.061
H1	+0.149	+0.178	+0.203	+0.062	+0.141	+0.157	+0.212	+0.055
H2	+0.133	+0.179	+0.216	+0.055	+0.111	+0.163	+0.207	+0.051
H3	+0.130	+0.156	+0.204	+0.055	+0.124	+0.160	+0.206	+0.050
H4	+0.120	+0.157	+0.203	+0.048	+0.122	+0.156	+0.207	+0.051
H5	+0.115	+0.155	+0.204	+0.046	+0.118	+0.161	+0.206	+0.051
H6	+0.126	+0.145	+0.204	+0.048	+0.130	+0.149	+0.206	+0.051
H7	+0.126	+0.145	+0.204	+0.049	+0.130	+0.149	+0.206	+0.051
H8	+0.126	+0.155	+0.204	+0.049	+0.118	+0.161	+0.206	+0.050
H9	+0.120	+0.157	+0.203	+0.048	+0.122	+0.156	+0.207	+0.051
H10	+0.130	+0.156	+0.204	+0.046	+0.124	+0.160	+0.206	–0.054
H11	+0.133	+0.179	+0.216	–0.064	+0.111	+0.163	+0.207	+0.047
H12	+0.149	+0.178	+0.203	+0.062	+0.141	+0.157	+0.212	+0.055

In bold: charge on nitrogen atom.

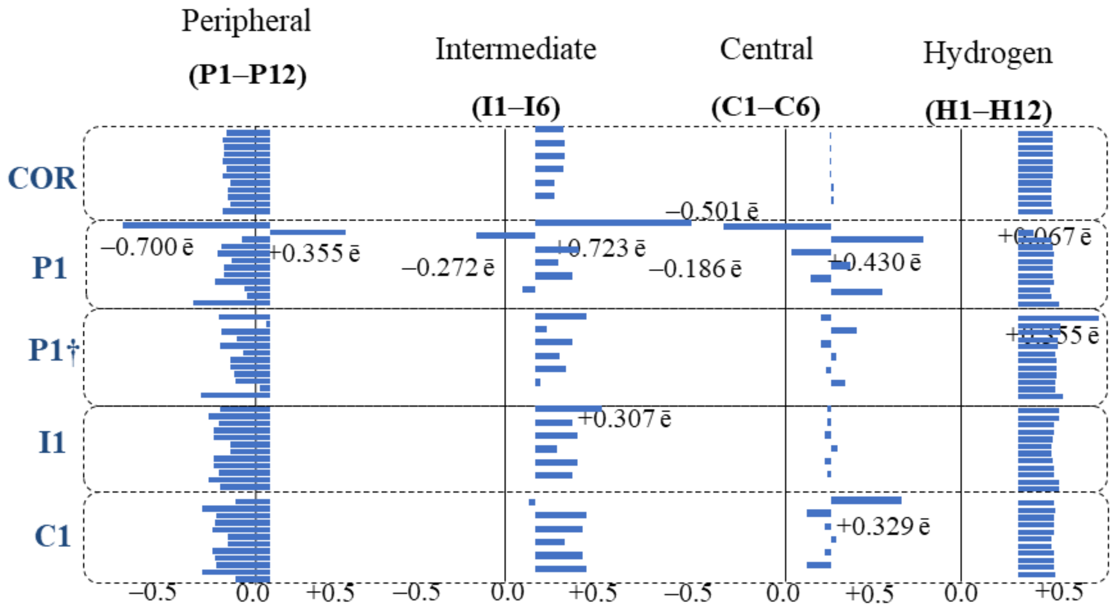

Scheme S1. MSK atomic charge distribution (in ē): on the coronene (COR) e N–coronene (P1, P1†, I1 and C1) units (in rows); on the Peripheral, Intermediate and Central regions (in collumns) and in hydrogen atoms (in collumn).

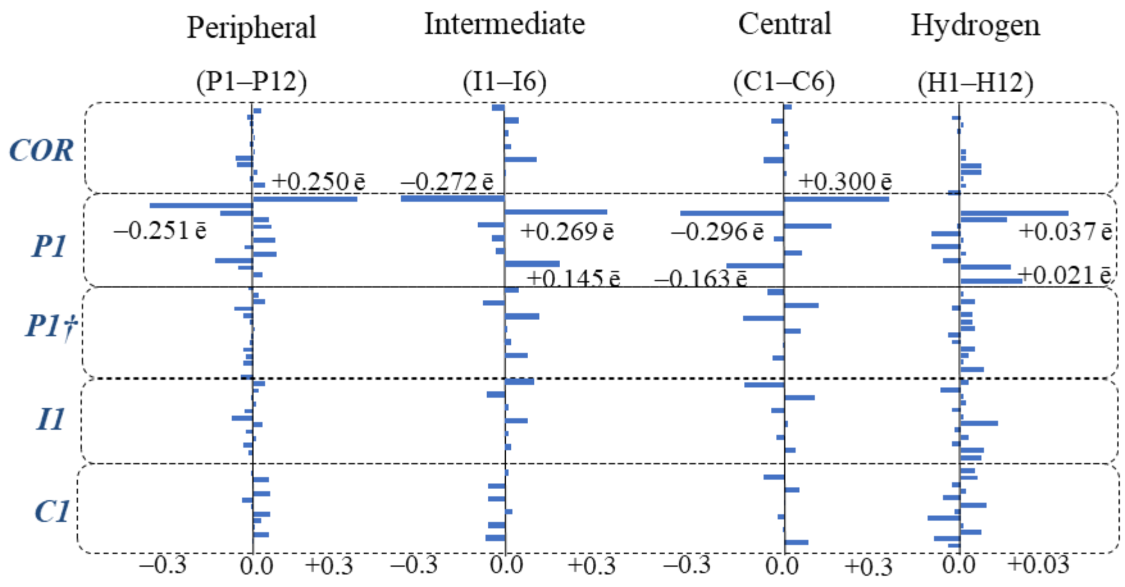

Scheme S2. MSK atomic charge variation distribution (in ē) in CO_2/coronene complexes. Total for the coronene (COR) e N–coronene (***P1, P1†, I1***, and ***C1***)/CO_2 complexes depicted in rows, while separated by regions (peripheral, intermediate, and central) and in hydrogen atoms, depicted in columns.

Table S6. MK atomic charge on the atoms of the coronene/CO_2 and N–coronene/CO_2 complexes in configurations of lowest energy.

Atom	***COR***	***P1***	***P1†***	***I1***	***C1***
P1	–0.191	**–0.450**	**–0.254**	–0.269	–0.166
P2	–0.241	+0.104	–0.003	–0.262	–0.333
P3	–0.231	–0.219	–0.201	–0.230	–0.215
P4	–0.225	–0.196	–0.208	–0.277	–0.261
P5	–0.222	–0.205	–0.261	–0.263	–0.235
P6	–0.221	–0.193	–0.136	–0.213	–0.232
P7	–0.224	–0.168	–0.185	–0.244	–0.212
P8	–0.232	–0.241	–0.192	–0.248	–0.236
P9	–0.241	–0.205	–0.184	–0.289	–0.240
P10	–0.191	–0.217	–0.188	–0.234	–0.250
P11	–0.197	–0.146	–0.072	–0.315	–0.289
P12	–0.198	–0.347	–0.353	–0.252	–0.163
I1	+0.092	+0.451	+0.272	**+0.385**	–0.024
I2	+0.171	–0.003	–0.005	+0.121	+0.191
I3	+0.146	+0.131	+0.261	+0.207	+0.174
I4	+0.144	+0.070	+0.116	+0.162	+0.152
I5	+0.172	+0.146	+0.153	+0.205	+0.175
I6	+0.091	+0.083	+0.084	+0.186	+0.184
C1	+0.019	–0.201	–0.097	–0.132	**+0.271**
C2	–0.041	+0.134	+0.219	+0.071	–0.072
C3	+0.008	–0.052	–0.165	–0.068	–0.025
C4	+0.011	+0.063	+0.072	+0.043	+0.008
C5	–0.043	–0.046	–0.026	–0.052	–0.032
C6	+0.021	+0.075	+0.034	+0.017	–0.047
H1	+0.149		+0.356	+0.181	+0.162
H2	+0.155	+0.104	+0.189	+0.172	+0.169
H3	+0.154	+0.162	+0.184	+0.157	+0.157
H4	+0.155	+0.151	+0.179	+0.159	+0.158
H5	+0.154	+0.150	+0.180	+0.152	+0.155
H6	+0.154	+0.153	+0.171	+0.146	+0.158
H7	+0.155	+0.145	+0.168	+0.158	+0.147
H8	+0.154	+0.157	+0.168	+0.153	+0.150
H9	+0.155	+0.152	+0.173	+0.160	+0.157
H10	+0.149	+0.156	+0.169	+0.153	+0.167
H11	+0.149	+0.146	+0.166	+0.187	+0.154
H12	+0.149	+0.199	+0.205	+0.185	+0.153
			CO_2 adsorbed		
C	+0.725	+0.634	+0.781	+0.620	+0.648
O	–0.363	–0.324	–0.400	–0.312	–0.333
O	–0.363	–0.320	–0.370	–0.318	–0.311

In bold: charge on nitrogen atom.

Table S7. CO_2 adsorption mode, interaction energy (Eint, in kcal mol-1), and CO_2–aromatic plane ($D_{CO2/plane}$) and CO_2–N (D_{CO2-N}) distances (in Å), in the CO_2/coronene and CO_2/N-coronene complexes, all optimized at the DFT level.

CO_2 ads.	Site	number (Fig. S2)	E_{int}	$D_{CO2/plane}$	D_{CO2-N}
COR	on-top	I	–3.08	3.25	
	Bridge	II	–3.33	3.18	
P1	Bridge	III	–3.24	3.19	4.80
	Bridge	IV	–3.03	3.26	6.17
	Bridge	V	–3.32	3.18	4.83
	H-bond	VI	–5.92	0.00	2.71
P1†	Bridge	VII	–3.65	3,20	4,56
	Bridge	VIII	–3.67	3,23	3,17
I1	on-top	IX	–3.56	3.18	4.07
	on-top	X	–3.05	3.16	5.68
	Bridge	XI	–3.72	3.09	3.10
	on-top	XII	–2.72	3.21	3.41
C1	Bridge	XIII	–3.57	3.23	3.17
	Bridge	XIV	–3.54	3.15	3.25
	Bridge	XV	–2.98	3.15	4.46
	Bridge	XVI	–3.16	3.19	3.11
	Bridge	XVII	–2.94	3.09	3.09

Table S8. Absolute Energy (in Hartree) and T1 diagnostic of the coronene and N-coronene isolated spicies.

Structures	Energy	T1 Diagnostic
COR	– 921.5876	0.009849940
P1	–937.6298	0.010262490
P1†	–935.6846	0.011566866
I1	–938.831	0.019636810
C1	–938.791	0.017780417

Table S9. Interaction energy (in kcal mol^{-1}) at Coupled Cluster level.

Structures	Energy
COR	–3,03
P1	–4,85
P1†	–3,13
I1	–3,60
C1	–3,34

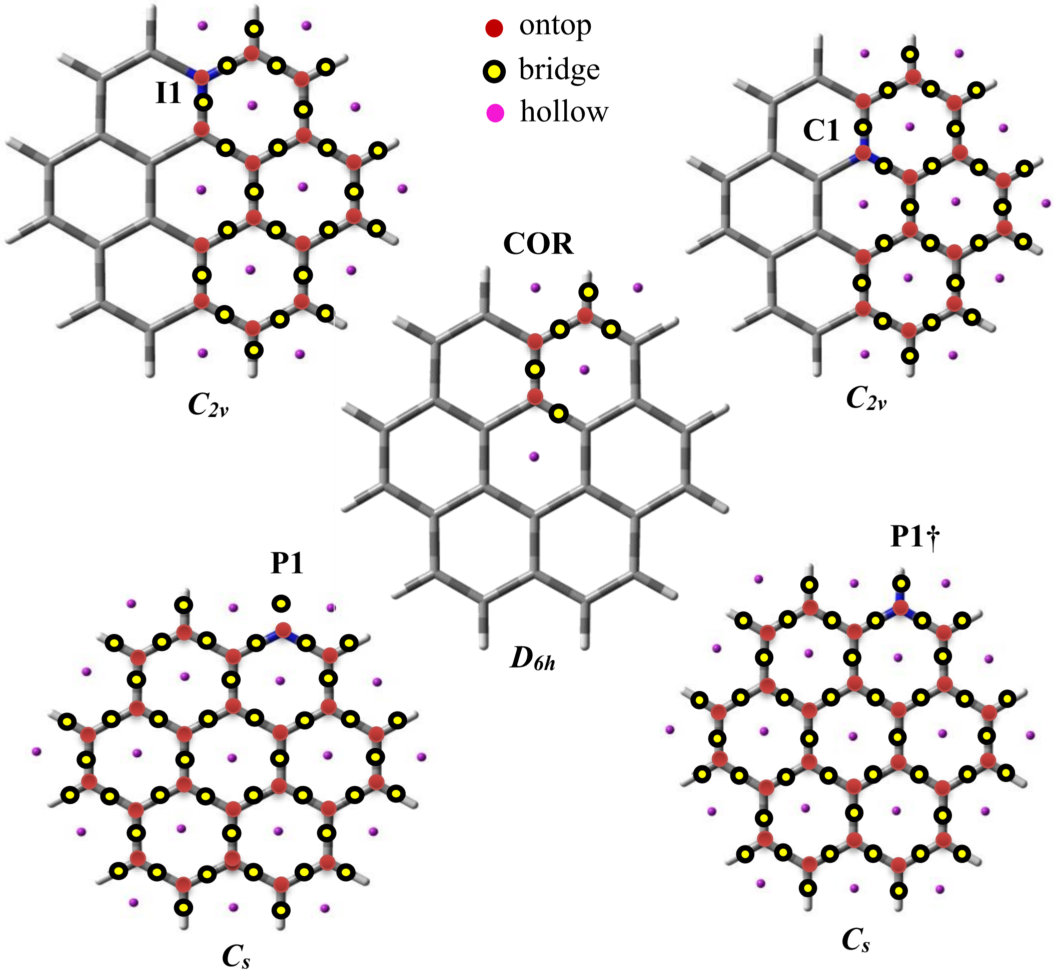

Figure S1. All CO_2 adsorption sites tested on the coronene (COR) and N–coronene (***P1, P1†, I1 e C1***) structures, considering the respective molecular symmetry.

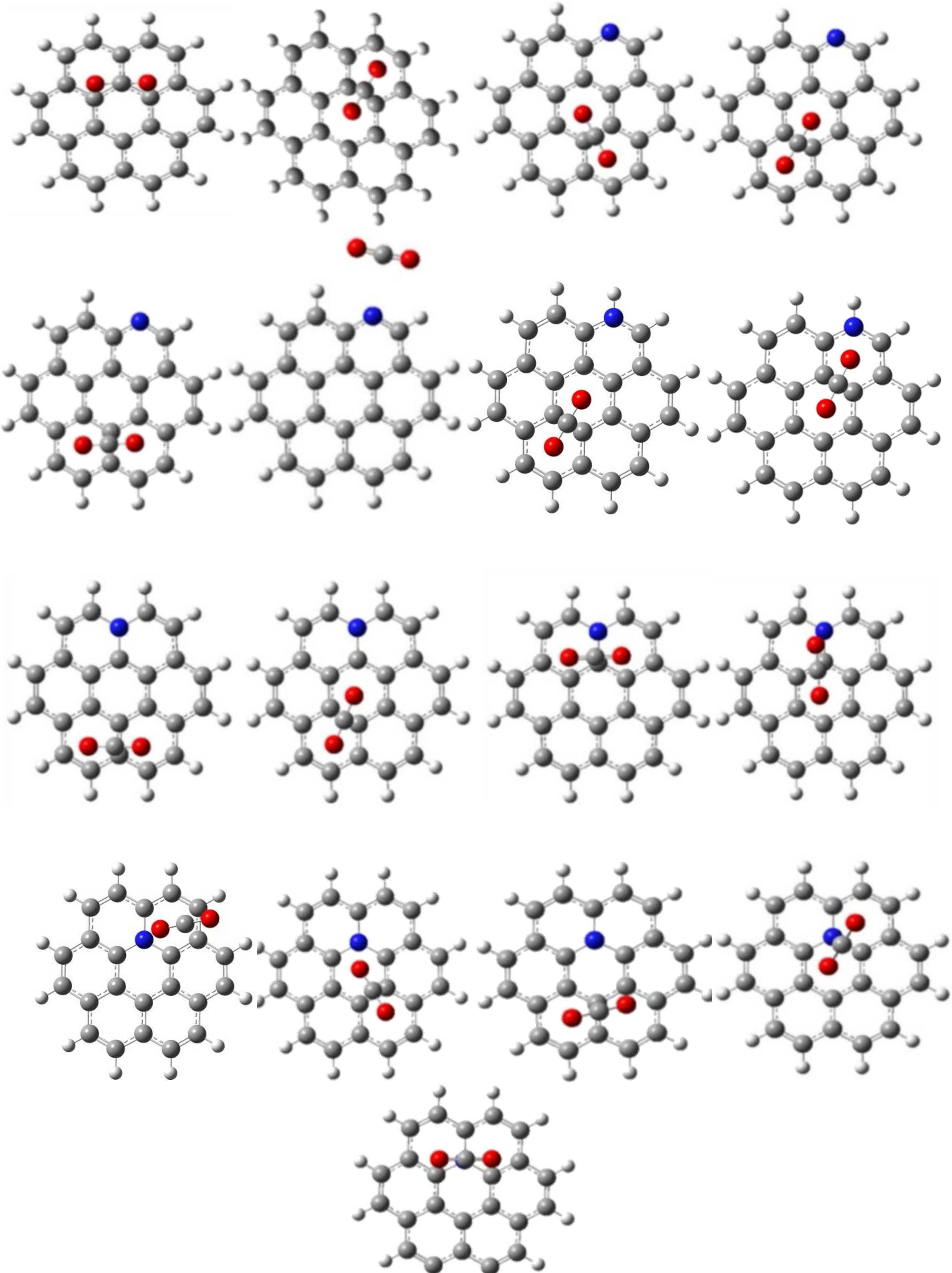

Figure S2. Geometries for all CO_2 configurations obtained on the coronene (COR) and N–coronene (***P1, P1†,I1*** e ***C1***) species.

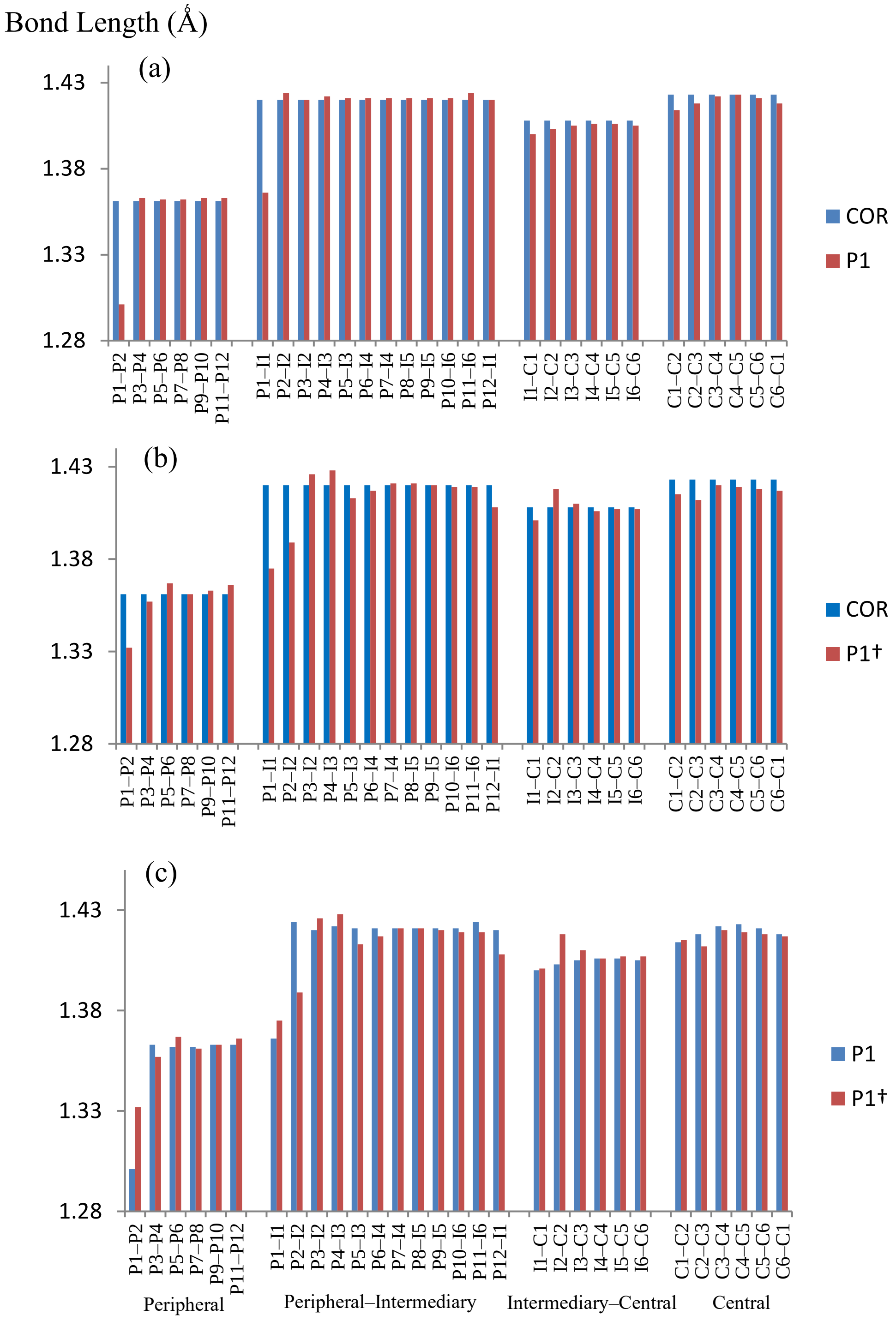

Scheme S3. C–C and C–N bond lengths (in Å) in the different regions in coronene and N-coronene (pyridinic–N): ***COR*** versus ***P1*** (a), ***COR*** versus ***P1†*** (b) and ***P1*** versus ***P1†*** (c).

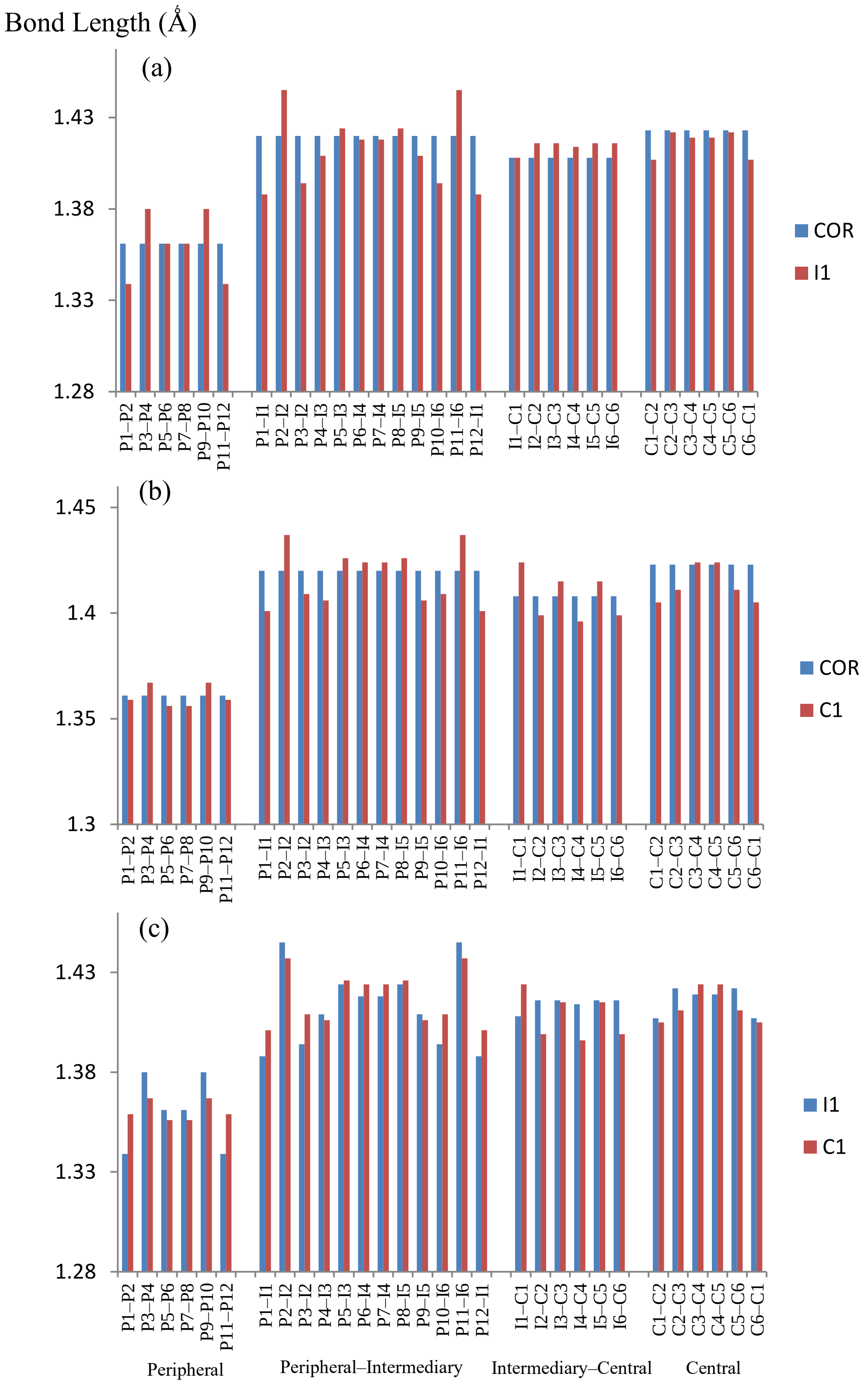

Scheme S4. C–C and C–N bond lengths (in Å) in the different regions in coronene and N-coronene (graphitic–N): ***COR*** versus ***I1*** (a), ***COR*** versus ***C1*** (b) and ***I1*** versus ***C1*** (c).
